# Biomarker profile and disease burden associated with intermittent and long-term oral corticosteroid use in patients with severe asthma prior to biologic initiation in real-life (STAR)

**DOI:** 10.1016/j.waojou.2025.101066

**Published:** 2025-06-03

**Authors:** Florence Schleich, Désirée Larenas-Linnemann, Alan Altraja, Luis Pérez de Llano, Konstantinos Kostikas, Mohsen Sadatsafavi, Arnaud Bourdin, Roy Alton Pleasants, Mark Hew, Wenjia Chen, Libardo Jiménez-Maldonado, Simon Couillard, Charlotte Suppli Ulrik, Adeeb A. Bulkhi, Ming-Ju Tsai, George C. Christoff, Nikolaos G. Papadopoulos, Paul E. Pfeffer, Dermot Ryan, Celine Bergeron, Mona S. Al-Ahmad, Delbert R. Dorscheid, Eileen Wang, John D. Blakey, Belinda Cochrane, Matthew J. Peters, Todor A. Popov, Carlos A. Torres-Duque, Susanne Hansen, Francesca Puggioni, Kirsty Fletton, Laila Salameh, Peter G. Middleton, Paulo Márcio Pitrez, Chin Kook Rhee, Eve Denton, Kenneth R. Chapman, Lauri Lehtimäki, Ruth B. Murray, Chau-Chyun Sheu, David J. Jackson, Riyad Al-Lehebi, Mariko Siyue Koh, Bassam Mahboub, Ledit R.F. Ardusso, Athena Gogali, Giorgio Walter Canonica, Piotr Kuna, Martin Sivori, Renaud Louis, Shelley Abercromby, Giuseppe Guida, Bernt Bøgvald Aarli, Aaron Beastall, Victoria Carter, Ghislaine Scelo, John Townend, Borja G. Cosio, Pujan H. Patel, Celine Yun Yi Goh, Zsuzsanna Csoma, John W. Upham, João A. Fonseca, Peter G. Gibson, Christine Jenkins, Guy G. Brusselle, Anne Chèvremont, Andréanne Côté, Carlos Andrés Celis-Preciado, Ivan Solarte, Celeste M. Porsbjerg, Asger Sverrild, Paula Kauppi, Stelios Loukides, Michael P. Makris, Andriana I. Papaioannou, Enrico Heffler, Jeffrey Shi Kai Chan, Hyonsoo Joo, Liam G. Heaney, Wei-Han Cheng, Njira Lugogo, Michael E. Wechsler, Cláudia Chaves Loureiro, Bellanid Rodríguez-Cáceres, Tatsuya Nagano, Zhixiao Wang, Hao-Chien Wang, Jorge Máspero, Fernando Saldarini, Ana María Stok, Anahi Yañez, Philip G. Bardin, Sinthia Z. Bosnic-Anticevich, Vidya Navaratnam, Mohit Bhutani, M. Diane Lougheed, Lyle Melenka, Petros Bakakos, Konstantinos P. Exarchos, Aggelos A. Ladias, Dóra Lúdvíksdóttir, Takashi Iwanaga, Elvia Angelica Contreras Contreras, Sverre Lehmann, José Alberto Ferreira, Rebecca Gall, Pin-Kuei Fu, Diahn-Warng Perng, Flavia Hoyte, Rohit Katial, Unnur S. Björnsdóttir, Camille Taillé, Christian Taube, Breda Cushen, Lakmini Bulathsinhala, Leif Bjermer, David B. Price, the ISAR STAR Working Group

**Affiliations:** a Department Pneumology CHU Liege, GIGA I3 Research Group, Exercise Physiology Lab, University of Liege, Liege, Belgium; b Centro de Excelencia en Asma y Alergia, Hospital Médica Sur, Ciudad de México, Mexico; c Department of Pulmonology, University of Tartu and Lung Clinic, Tartu University Hospital, Tartu, Estonia; d Pneumology Service. Lucus Augusti University Hospital. EOXI (Sergas (Galician Healthcare Service) Integrated Management Structure): Lugo, Monforte, Cervo, Spain; e Department of Psychiatry, Radiology, Public Health, Nursery and Medicine, University of Santiago de Compostela, Santiago de Compostela, Spain; f Respiratory Medicine Department, University of Ioannina School of Medicine, Ioannina, Greece; g Respiratory Evaluation Sciences Program, Faculty of Pharmaceutical Sciences, The University of British Columbia, Vancouver, British Columbia, Canada; h PhyMedExp, Univ Montpellier, CNRS (National Center for Scientific Research), INSERM (The National Institute of Health and Medical Research), CHU (Centre Hospitalier Universitaire) Montpellier, Montpellier, France; i Marsico Lung Institute, University of North Carolina, Chapel Hill, NC, USA; j Department of Medicine, Division of Pulmonary and Critical Care Medicine, University of Michigan, Ann Arbor, MI, USA; k Allergy, Asthma & Clinical Immunology, Alfred Health, Melbourne, Victoria, Australia; l Public Health and Preventive Medicine, Monash University, Melbourne, Victoria, Australia; m Saw Swee Hock School of Public Health, National University of Singapore, Singapore; n Fundación Neumológica Colombiana, ASMAIRE REXPIRA Program, Bogotá, Colombia; o Departamento Cundinamarca, Universidad de La Sabana, Chia, Colombia; p Faculté de médecine et des sciences de la santé, Université de Sherbrooke, Sherbrooke, Canada; q Department of Respiratory Medicine, Copenhagen University Hospital - Hvidovre, Copenhagen, Denmark; r Department of Medicine, College of Medicine, Umm Al-Qura University, Makkah, Saudi Arabia; s Division of Pulmonary and Critical Care Medicine, Department of Internal Medicine, Kaohsiung Medical University Hospital, Kaohsiung Medical University, Kaohsiung, Taiwan; t Department of Internal Medicine, School of Medicine, College of Medicine, Kaohsiung Medical University, Kaohsiung, Taiwan; u Faculty of Public Health, Medical University, Sofia, Bulgaria; v Division of Infection, Immunity & Respiratory Medicine, University of Manchester, Manchester, UK; w Allergy Department, 2nd Pediatric Clinic, University of Athens, Athens, Greece; x Department of Respiratory Medicine, Barts Health NHS (National Health Services) Trust, London, UK; y Barts and the London School of Medicine and Dentistry, Queen Mary University of London, London, UK; z Usher Institute, University of Edinburgh, Edinburgh, UK; aa Department of Medicine, Centre for Lung Health, Vancouver General Hospital, Vancouver, British Columbia, Canada; ab Department of Medicine, The University of British Columbia, Vancouver, British Columbia, Canada; ac Microbiology Department, College of Medicine, Kuwait University, Kuwait City, Kuwait; ad Al-Rashed Allergy Center, Ministry of Health, Kuwait City, Kuwait; ae Center for Heart Lung Innovation, The University of British Columbia, Vancouver, Canada; af Division of Allergy and Clinical Immunology, Department of Medicine, National Jewish Health, Denver, CO, USA; ag Division of Allergy and Clinical Immunology, Department of Medicine, University of Colorado School of Medicine, Aurora, CO, USA; ah Respiratory Medicine, Sir Charles Gairdner Hospital, Perth, Australia; ai Curtin University Medical School, Perth, Australia; aj Department of Respiratory and Sleep Medicine, Campbelltown Hospital (SWSLHD), Campbelltown, New South Wales, Australia; ak School of Medicine, Western Sydney University, Sydney, New South Wales, Australia; al Department of Thoracic Medicine, Concord Hospital, Sydney, New South Wales, Australia; am Macquarie Medical School, Faculty of Medicine, Health and Human Sciences, Macquarie University, Sydney, New South Wales, Australia; an University Hospital St. Ivan Rilski, Sofia, Bulgaria; ao CINEUMO (Centro Internacional de Investigación en Neumología), Respiratory Research Center, Fundación Neumológica Colombiana, Bogotá, Colombia; ap Respiratory Research Unit, Bispebjerg University Hospital, Copenhagen, Denmark; aq Center for Clinical Research and Prevention, Bispebjerg and Frederiksberg Hospital, Copenhagen, Denmark; ar Personalized Medicine, Asthma and Allergy Clinic, IRCCS Humanitas Research Hospital, Rozzano MI, Italy; as Observational and Pragmatic Research Institute, Singapore, Singapore; at Optimum Patient Care Global, Cambridge, UK; au Rashid Hospital, Dubai Health (DH), Dubai, United Arab Emirates; av Medical Education and Research Department, Dubai Health, Dubai, United Arab Emirates; aw CITRICA, Dept Resiratory & Sleep Medicine, Westmead Hospital, Sydney, Australia; ax Pulmonology Division, Hospital Santa Casa de Porto Alegre, Porto Alegre, Brazil; ay Division of Pulmonary and Critical Care Medicine, Department of Internal Medicine, Seoul St. Mary's Hospital, College of Medicine, The Catholic University of Korea, Seoul, South Korea; az Department of Medicine, Central Clinical School, Monash University, Clayton, Melbourne, Victoria, Australia; ba University of Toronto, Toronto, Ontario, Canada; bb Allergy Centre, Tampere University Hospital, Tampere, Finland; bc Faculty of Medicine and Health Technology, Tampere University, Tampere, Finland; bd Guy's Severe Asthma Centre, School of Immunology & Microbial Sciences, King's College London, London, UK; be Department of Pulmonology, King Fahad Medical City, Riyadh, Saudi Arabia; bf College of Medicine, Alfaisal University, Riyadh, Saudi Arabia; bg Department of Respiratory and Critical Care Medicine, Singapore General Hospital, Singapore, Singapore; bh College of Medicine, University of Sharjah, Sharjah, United Arab Emirates; bi Pulmonology, Allergy and Immunology Department, Rosario School of Medicine, National University of Rosario, Rosario, SF, Argentina; bj Department of Biomedical Sciences, Humanitas University, Pieve Emanuele, Milan, Italy; bk Division of Internal Medicine Asthma and Allergy, Medical University of Lodz, Lodz, Poland; bl Neumonology Center of School of Medicine, University of Buenos Aires, Hospital “Dr.JM.Ramos Mejia”, Buenos Aires, Argentina; bm The Centre for Lung Health, Vancouver Coastal Health Research Institute, UBC, Vancouver, Canada; bn Department of Clinical and Biological Sciences, Severe Asthma and Rare Lung Disease Unit, San Luigi Gonzaga University Hospital, University of Turin, Orbassano, Turin, TO, Italy; bo Department of Thoracic Medicine, Haukeland University Hospital, Bergen, Norway; bp Department of Clinical Science, University of Bergen, Bergen, Norway; bq Son Espases University Hospital-IdISBa (Institut d’Investigació Sanitària Illes Balears)-Ciberes, Mallorca, Spain; br Respiratory Medicine, Royal Brompton Hospital, London, UK; bs Asthma Outpatient Clinic, National Koranyi Institute for Pulmonology, Budapest, Hungary; bt Frazer Institute & PA-Southside Clinical Unit, The University of Queensland, Brisbane, Queensland, Australia; bu CINTESIS@RISE (Center for Health Technology and Services Research at Health Research Network), MEDCIDS (Departamento Medicina da Comunidade, Informação e Decisão em Saúde/Department of Community Medicine, Information and Health Decisions), Faculty of Medicine of the University of Porto, Porto, Portugal; bv Australian Severe Asthma Network, Priority Research Centre for Healthy Lungs, University of Newcastle, Newcastle, New South Wales, Australia; bw Hunter Medical Research Institute, Department of Respiratory and Sleep Medicine, John Hunter Hospital, New Lambton Heights, New South Wales, Australia; bx Respiratory Medicine, UNSW, Sydney, Head Respiratory Group, George Institute, Australia; by Department of Respiratory Medicine, Ghent University Hospital, Ghent, Belgium; bz Departments of Epidemiology and Respiratory Medicine, Erasmus Medical Center Rotterdam, Rotterdam, the Netherlands; ca CHU Sart-Tilman Liège, 4000 Liège, Belgium; cb Department of Medicine, Laval University, Quebec City, Quebec, Canada; cc Pulmonary Unit, Hospital Universitario San Ignacio, Bogota, Colombia; cd Faculty of Medicine, Pontificia Universidad Javeriana, Bogota, Colombia; ce Department of Respiratory Medicine and Infectious Diseases, Research Unit, Bispebjerg Hospital, Copenhagen, Denmark; cf Department of Clinical Medicine, University of Copenhagen, Copenhagen, Denmark; cg The Heart and Lung Center, Helsinki, University Hospital, University of Helsinki, Helsinki, Finland; ch 2nd Respiratory Medicine Department, Attikon University Hospital, University of Athens Medical School, Athens, Greece; ci Allergy Unit, National and Kapodistrian University of Athens, University General Hospital ‘Attikon’, Greece; cj Division of Pulmonary and Critical Care Medicine, Department of Internal Medicine, Uijeongbu St. Mary's Hospital, College of Medicine, The Catholic University of Korea, Seoul, South Korea; ck Wellcome-Wolfson Institute for Experimental Medicine, Queen's University Belfast, Belfast, UK; cl Sanofi, Cambridge, MA, USA; cm National Jewish Health Cohen Family Asthma Institute, Department of Medicine, National Jewish Health, Denver, CO, USA; cn Pneumology Unit, Hospitais da Universidade de Coimbra, Centro Hospitalar e Universitário de Coimbra, Coimbra, Portugal; co Centre of Pneumology, Faculty of Medicine, University of Coimbra, Coimbra, Portugal; cp Instituto Neumológico del Oriente, Bucaramanga, Colombia; cq Division of Respiratory Medicine, Department of Internal Medicine, Kobe University Graduate School of Medicine, Kobe, Japan; cr Regeneron Pharmaceuticals, Inc., Tarrytown, NY, USA; cs Department of Medicine, Cancer Center, National Taiwan University, Taipei, Taiwan; ct Clinical Research for Allergy and Respiratory Medicine, CIDEA Foundation, Buenos Aires, Argentina; cu University Career of Specialists in Allergy and Clinical Immunology at the Buenos Aires University School of Medicine, Buenos Aires, Argentina; cv Section Pulmonology, Hospital Santojanni Autonomous, City of Buenos Aires, Argentina; cw Investigaciones en Patologias Respiratorias, San Miguel de Tucuman, Tucuman, Argentina; cx The Allergy and Respiratory Medicine Research Center- InAER – Buenos Aires, Argentina; cy Lung, Sleep, Allergy and Immunology, Monash Health Clayton, Monash University, Clayton, Melbourne, Victoria, Australia; cz AstraZeneca Pty Ltd, Medical Affairs, Biopharmaceuticals Unit, Sydney, New South Wales, Australia; da Woolcock Institute of Medical Research, Sydney, New South Wales, Australia; db The University of Western Australia, Perth, Australia; dc Division of Pulmonary Medicine, Department of Medicine, University of Alberta, Edmonton, Alberta, Canada; dd Asthma Research Unit, Kingston General Hospital Research Institute, Kingston, ON, Canada; de Department of Medicine, Queen's University, Kingston, ON, Canada; df Department of Public Health Sciences, Queen's University, Kingston, ON, Canada; dg Synergy Respiratory Care, Sherwood Park, Canada; dh 1st Department of Respiratory Medicine, National and Kapodistrian University of Athens, Athens, Greece; di Pulmonology Resident in the University Hospital of Ioannina, Ioannina, Greece; dj Department of Respiratory Medicine, Landspitali University Hospital Reykjavik Iceland, University of Iceland, Reykjavik, Iceland; dk Kindai University Hospital, Osakasayama, Japan; dl Mexican Council of Clinical Immunology and Allergy, Mexico City, Mexico; dm Lic. Adolfo López Mateos Regional Hospital of the Institute of Security and Social Services for State Workers (ISSSTE), Mexico City, Mexico; dn Allergy Department - ULS Gaia e Espinho, Vila Nova de Gaia, Portugal; do Department of Medical Research, Taichung Veterans General Hospital, Taichung, Taiwan; dp School of Medicine, National Yang Ming Chiao Tung University, Taipei, Taiwan; dq Department of Chest Medicine, Taipei Veterans General Hospital, Taipei, Taiwan; dr Department of Allergy and Respiratory Medicine, University Hospital, Reykjavik, Iceland; ds Department of Respiratory Diseases, Bichat Hospital, Assistance publique – Hôpitaux de Paris), Université Paris Cité, Paris, France; dt Department of Pulmonary Medicine, University Medical Center Essen-Ruhrlandklinik, Essen, Germany; du Department of Respiratory Medicine, Beaumont Hospital, Dublin, Ireland; dv Respiratory Medicine and Allergology, Department of Clinical Sciences, Skåne University Hospital, Lund University, Lund, Sweden; dw Centre of Academic Primary Care, Division of Applied Health Sciences, University of Aberdeen, Aberdeen, UK

**Keywords:** BEC, Blood eosinophil count, Fractional exhaled nitric oxide, FeNO, IgE, Immunoglobulin E, Intermittent, Long-term

## Abstract

**Background:**

Asthma characterization using blood eosinophil count (BEC) (among other biomarkers and clinical indices) is recommended in severe asthma (SA), but the masking effect of oral corticosteroids (OCS), makes this challenging.

**Aim:**

Our aim was to explore the effect of OCS use (both intermittent [iOCS] and long-term [LTOCS]) prior to biologic initiation on SA phenotype and biomarker profile in real-life and to characterize the burden of SA among patients prescribed LTOCS by biomarker profile.

**Methods:**

This was a registry-based cohort study, including data from 23 countries collected between 2003 and 2023 and shared with the Internatonal Severe Asthma Registry (ISAR). Patients with SA were categorized into 3 cohorts, those with: (i) no prescription for OCS, (ii) prescription(s) for iOCS (ie, ≤90 days in previous 12-months, usually short courses for exacerbations), and (iii) prescriptions for LTOCS (ie, >90 days in previous 12-months). Biomarker distribution (ie, BEC, fractional exhaled nitric oxide [FeNO], and total Immunoglobulin E [IgE]) were quantified in the year prior to biologic initiation in patients with SA according to OCS prescription pattern. Phenotypes were characterized for those prescribed LTOCS according to BEC cut-off (<150 and ≥ 150 cells/μL).

**Results:**

Of 4305 patients included, 5.0% (n = 215), 54.1% (n = 2330) and 40.9% (n = 1760) were prescribed no OCS, iOCS, and LTOCS, respectively. The BEC distribution varied by prescription pattern and LTOCS dose (<5 mg to ≥20 mg/day); BEC was <150 cells/μL in 28.6% (n = 369/1288) of LTOCS patients, compared to 19.5% (n = 284/1460) of iOCS patients and 14.0% (n = 21/150) of those in the no OCS group. Median BEC was also significantly lower in the LTOCS versus the iOCS group (310 vs 400 cells/μL; p < 0.001). A similar pattern was noted for IgE, but not FeNO. Among LTOCS patients with BEC <150 cells/μL, 39.9% experienced ≥4 exacerbations, 75.1% had uncontrolled asthma symptoms and 55.9% had evidence of persistent airflow obstruction (compared with 40.9%, 76.2% and 59.5% of those with BEC ≥150 cells/μL, respectively).

**Conclusions:**

OCS, whether prescribed intermittently or long term, affect BEC distribution potentially leading to heightened risk of phenotype misclassification and influencing subsequent treatment decisions. FeNO appears to be less susceptible to OCS-induced suppression. Disease burden was high for those in the LTOCS group and was high independent of dose and BEC. Our findings highlight the importance of considering OCS use, even intermittent use, when characterizing SA, and suggests the need for earlier phenotyping and alternative treatment strategies for LTOCS patients with low BEC.

## Introduction

Asthma is a heterogenous condition, with diverse phenotypes and endotypes.^1^, ^2^, ^3^ The type 2 (T2)-inflammatory endotype is associated with elevated fractional exhaled nitric oxide (FeNO) concentrations and increased blood eosinophil count (BEC), and categorized along a continuum of T2 involvement from T2-high to T2-low.^3^ The T2-high endotype is the most common in patients with severe asthma (SA), accounting for over 80% of adults by recent estimates.^3^^,^^4^ Higher BEC and/or FeNO predict exacerbation risk,^5^^,^^6^ healthcare resource utilisation (HCRU),^7^ accelerated lung function decline,^8^ as well as response to biologics and inhaled corticosteroids (ICS),^9^^,^^10^ and are thus important clinical metrics to inform treatment decisions and facilitate precision medicine in SA management. The Global Initiative for Asthma (GINA) recommends phenotyping at Step 5 and characterizes T2-disease as BEC ≥150 cells/μL and/or FeNO ≥20 ppb, and/or evidence, which is clinically allergen-driven.^3^

First reported by Morrow-Brown back in 1958,^11^ it is now well-established that oral corticosteroids (OCS) have limited utility in the absence of T2/eosinophilic inflammation.^3^^,^^12^^,^^13^ However, in the absence of alternatives, GINA still recommends consideration of long-term oral corticosteroids (LTOCS) at Step 5 as a last resort for those with poor symptom control and/or frequent exacerbations despite good inhaler technique, adherence to treatment, and after exclusion of contributory factors and a trial of other add-on treatments, including biologics (where available and affordable).^3^ This GINA recommendation may explain the high prevalence of LTOCS use in those with SA,^14^ despite their well-documented cumulative side effects, including obesity, diabetes, osteoporosis, adrenal suppression, hypertension, eye issues such as cataract or glaucoma, and psychologic side effects such as depression and anxiety and psychiatric disorders.^12^^,^^13^^,^^15^^,^^16^ Even short-term OCS use, as few as 4 lifetime courses, is associated with adverse effects.^17^, ^18^, ^19^ Patient and health-care provider education on the potential risks of both short- and long-term OCS use is urgently needed. Indeed, a call to action endorsed by both the World Allergy Organization and the Respiratory Effectiveness Group advocates for the implementation of OCS-sparing strategies and development of OCS stewardship initiatives, with the literature supporting an OCS cumulative dose threshold of 0.5–1 g/year (prednisolone equivalent).^12^

An inverse relationship exists between current OCS dose and BEC in patients with severe eosinophilic asthma,^20^, ^21^, ^22^ leading to the potential for mis-classification as T2-low asthma in the presence of OCS (and also high dose ICS).^23^^,^^24^ This issue is recognized by GINA which recommends repeat biomarker profiling (up to 3 times) and at least 1–2 weeks after OCS use or on the lowest possible OCS dose,^3^ although others have cautioned that levels may take longer to rebound after OCS discontinuation.^25^ The impact of LTOCS use on BEC is also considered when grading likelihood of eosinophilic phenotype in the gradient algorithm proposed by Heaney et al.^4^ However, the extent and variability of BEC suppression in response to OCS is unknown and might vary amongst individuals. The potential masking effect of OCS on BEC is particularly pertinent in SA, where OCS use is prevalent^14^ and where their use is often stipulated as a pre-requisite for biologic prescription in many countries.^26^ Some countries and individual payers preclude biologic use in those with lower BEC, even when receiving LTOCS.^26^ Whether intermittent OCS (iOCS) versus LTOCS use have similar or different differential effects on BEC (and other biomarkers) merits further study, since iOCS use is often considered by physicians as less harmful.^19^ Approximately 50% of patients with SA treated at GINA Step 5 use OCS intermittently.^14^

A low BEC does not necessarily rule out the presence of eosinophilia,^27^ and if used in isolation as an indicator of airway eosinophilia it may lead to a substantial number of false negatives.^21^^,^^28^ Unfortunately, a low BEC also does not rule out the presence of significant morbidity. Previous research has shown a high burden of SA both with and without elevated T2-inflammatory biomarkers.^29^, ^30^, ^31^ These patients have fewer treatment options available to them and may actually benefit from reduction in some medication classes — T2-low disease is often over-treated with corticosteroids.^32^ However, the relative disease burden among LTOCS users with high BEC cut-off (who may qualify for biologic treatment) versus those with low BEC cut-off (who may not qualify for biologic treatment) is under-studied. Our aim was to explore the effect of OCS prescription (both intermittent and long-term) prior to biologic initiation on SA phenotype and biomarker profile in real-life and to characterize the burden of disease among LTOCS patients by biomarker profile.

## Methods

### Study design and data source

This was an historical cohort study using data from the International Severe Asthma Registry (ISAR; https://www.isar.opcglobal.org/), the largest adult SA registry in the world, currently holding data on >20,000 patients from 29 countries. Patients with SA included in ISAR have been well characterized,^14^ phenotyped,^4^ and endotyped.^33^^,^^34^ The details of this registry have been described elsewhere (and also in the online supplement).^35^ Here, we have included data from 23 countries (Argentina (n = 12), Australia (n = 130), Belgium (n = 180), Bulgaria (n = 29), Canada (n = 163), Colombia (n = 30), Denmark (n = 611), Greece (n = 66), India (n = 3), Italy (n = 704), Japan (n = 32), Korea (n = 25), Kuwait (n = 146), Mexico (n = 45), Poland (n = 121), Portugal (n = 9), Saudi Arabia (n = 39), Singapore (n = 7), Spain (n = 195), Taiwan (n = 40), United Arab Emirates (UAE; n = 36), United Kingdom (UK; n = 758), and United States of America (USA; n = 924)) which shared data with ISAR. The Index date corresponded to date of biologic initiation. SA patients were characterized by OCS use (none, intermittent, and long-term), and LTOCS users subsequently characterized according to BEC cut-off (i.e. < or ≥150 cells/μL). All data were pre-biologic initiation.

### Ethics and registration

This study fulfilled requirements outlined in the REal Life Evidence AssessmeNt Tool recommendations for comparative effectiveness research studies including a *priori* development of both protocol and statistical analysis plan,^36^ and complied with appropriate registration and ethics requirements. The study was registered with the European Network Centres for Pharmacoepidemiology and Pharmacovigilance (ENCePP; EUPAS 49201), and designed, implemented, and reported in compliance with the ENCePP and with all applicable local and international laws and regulations. Ethics approval was obtained from the Anonymized Data Ethics Protocols and Transparency Committee (ADEPT1022).

### Patients

All patients were enrolled into ISAR and were required to be ≥ 18 years of age at date of biologic initiation, have SA (consistently defined as receiving treatment at GINA 2018 Step 5 or with uncontrolled asthma at GINA Step 4),^37^ to have initiated biologic therapy, and have registry data for ≥1 year prior to subsequent biologic initiation.

Patients were categorized into 3 cohorts, those with (i) no prescriptions for OCS, (ii) prescription(s) for iOCS (ie, ≤90 days, usually short courses of rescue steroids for the treatment of exacerbations), and (iii) prescriptions for LTOCS (ie, >90 days) in the 12 months prior to biologic initiation. Those in the LTOCS group included in the characterization by BEC cut-off analysis were also required to have a BEC reading (most recent at or prior to biologic initiation; S-Table 1). Those, who had received bronchial thermoplasty, had missing biologic initiation date, those with no pre-biologic assessment or those with a co-morbidity, whose treatment guidelines conventionally include LTOCS (eg, allergic bronchopulmonary aspergillosis, vasculitis, inflammatory bowel disease, immune deficiency disorder), were excluded.

### Variables

Demographic, biomarker, and disease characteristic variables were collected (S-Table 2). BEC, FeNO and total serum Immunoglobulin E (IgE) concentrations were the most recent values recorded in the 1-year period prior to biologic initiation, a median of 0–24 days, 0–5 days and 5–18 days for BEC, FeNO, and IgE, prior to biologic initiation, respectively (S-Table 1)**.** Recorded disease characteristics were asthma onset and duration, eosinophilic phenotype^4^ (S-Table 3)**,** exacerbations, asthma control, lung function, asthma treatment pattern, and healthcare resource utilization (HCRU) – ie, asthma-related emergency department (ED) visit or hospitalization. An exacerbation was defined as an asthma-related hospital attendance/admission and/or an asthma related emergency room attendance, and/or an acute OCS course of ≥3 days. Asthma control was categorized as well controlled, partly controlled, or uncontrolled according to GINA 2022 criteria,^38^ Asthma Control Test (ACT), or Asthma Control Questionnaire (ACQ). Lung function was assessed using percent predicted forced expiratory volume in 1 s (ppFEV_1_) and FEV_1_/forced vital capacity (FVC) and eosinophilic gradient phenotype defined as per Heaney et al (see online supplement).^4^

### Statistics

Descriptive statistical analyses were pre-defined and STATA version 18 and R 4.2.0 (Tx, USA) were used to conduct all statistical analyses. Continuous variables were summarized using means, standard deviations (SD), medians, and interquartile ranges. Categorical variables were summarized as n (%). Comparisons between groups were carried out using t-tests (for normally distributed variables), Mann-Whitney tests (for non-normally distributed continuous variables), Poisson regression for exacerbations, or chi-square tests (for categorical variables), as appropriate. Patients were included in all analyses for which relevant data were available. Numbers of patients with non-missing data for each of the variables are reported in the tables. All statistical analyses were exploratory with P-values ≤0.05 considered to be statistically significant.

## Results

### Patient disposition

**Fig. 1:**
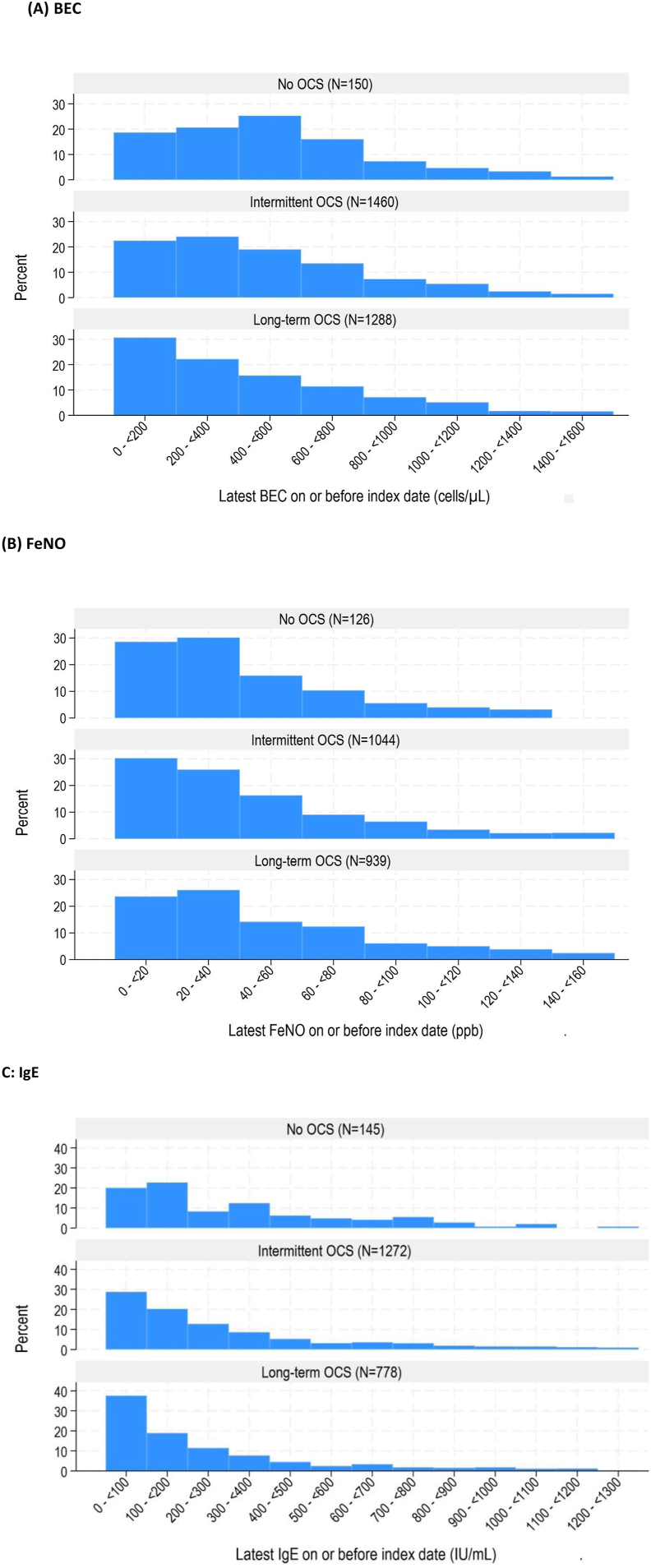
Pre-biologic (A) BEC, (B) FeNO, and (C) IgE distribution according to OCS use for patients with severe asthma. A leftward shift in the BEC distribution was observed in the intermittent and, especially, in the long-term OCS groups compared to no OCS users (A). The impact of OCS prescription was less apparent for FeNO (B). The IgE concentration was significantly lower in patients long-term OCS compared to those, who were prescribed intermittent OCS (p < 0.001) (C). BEC: blood eosinophil count; FeNO: fractional exhaled nitric oxide; IgE: Immunoglobulin E; OCS: oral corticosteroid; Intermittent use: ≤90 days; Long-term use: >90 days; Index date: date of biologic initiation

As of October 2023, ISAR included data on 17,553 adult patients with SA, of whom 9014 (51.4%) received biologics (S-Fig. 1). The remaining exclusions were predominantly due to insufficient data to determine their pre-biologic OCS use (n = 3737; 21.3%). In total, 4,305 patients from 23 countries were eligible for inclusion in the present study.

### Overall phenotypic characterization

The majority of patients in the final cohort were female (61% (n = 2624/4304)), aged >50 years upon subsequent biologic initiation, overweight/obese (69.9%; n = 2688/3846), never smokers (66.3%; n = 2406/3627), had an eosinophilic phenotype^4^ (88.8%; n = 3285/3698) and adult-onset disease (69.5%; n = 1917/2759) (Table 1). The majority had ≥2 exacerbations in the previous year (63.6%; n = 2561/4030), uncontrolled disease (71.9%; n = 2022/2813), and evidence of persistent airflow obstruction (53.4%; n = 1700/3184) (Table 1). OCS burden was also high, and anti-IL5/5R was the most common subsequently prescribed biologic (61.5%). Omalizumab was the most commonly prescribed biologic pre-2017 – see S-Table 4.

**Table 1:** Demographic and clinical characteristics plus HCRU of severe asthma patients pre-biologic initiation by OCS treatment pattern.

	No OCS N = 215	iOCS^a^ N = 2330	LTOCS^a^ N = 1760	Total N = 4305	iOCS vs LTOCS P-value
**Demographic characteristics**					
**Sex**		N = 2329		N = 4304	
Female, n (%)	130 (60.5)	1424 (61.1)	1070 (60.8)	2624 (61.0)	0.822
**Age at biologic initiation**					
Mean (SD)	52.5 (14.2)	51.9 (14.5)	53.0 (14.1)	52.4 (14.3)	0.011
**Age at biologic initiation, yrs**					
18-34, n (%)	26 (12.1)	323 (13.9)	212 (12.0)	561 (13.0)	
35-54, n (%)	83 (38.6)	936 (40.2)	668 (38.0)	1687 (39.2)	0.043
55-79, n (%)	104 (48.4)	1050 (45.1)	857 (48.7)	2011 (46.7)	
≥80, n (%)	2 (0.9)	21 (0.9)	23 (1.3)	46 (1.1)	
**BMI, kg/m^2^**	N = 186	N = 2102	N = 1558	N = 3846	
Median (IQR)	27 (24–31)	28 (24–32)	28 (24–33)	28 (24–32)	0.732
Underweight, n (%)	3 (1.6)	33 (1.6)	35 (2.2)	71 (1.8)	0.106
Normal, n (%)	61 (32.8)	581 (27.6)	445 (28.6)	1087 (28.3)	
Overweight, n (%)	71 (38.2)	714 (34.0)	478 (30.7)	1263 (32.8)	
Obese (≥30), n (%)	51 (27.4)	774 (36.8)	600 (38.5)	1425 (37.1)	
**Smoking status**	N = 183	N = 1976	N = 1468	N = 3627	
Current smoker, n (%)	7 (3.8)	62 (3.1)	41 (2.8)	110 (3.0)	0.523
Ex-smoker, n (%)	48 (26.2)	596 (30.2)	467 (31.8)	1111 (30.6)	
Never smoked, n (%)	128 (69.9)	1318 (66.7)	960 (65.4)	2406 (66.3)	
**Biomarkers**					
**BEC cells/μL**	N = 150	N = 1460	N = 1288	N = 2898	
Mean (SD)	569.9 (615.3)	531.5 (525.7)	490.4 (547.0)	515.2 (540.5)	
Median (IQR)	460 (210–700)	400 (200–700)	310 (100–695)	400 (180–700)	<0.001
**FeNO (ppb)**	N = 126	N = 1044	N = 939	N = 2109	
Mean (SD)	45.3 (42.9)	49.3 (47.1)	58.0 (54.5)	53.0 (50.5)	
Median (IQR)	36 (17–61)	34 (17–65)	40 (20–77)	36 (18–70)	<0.001
**IgE, IU/ml**	N = 145	N = 1272	N = 778	N = 2195	
Mean (SD)	530.8 (796.5)	494.7 (1747.6)	397.2 (818.9)	462.5 (1432.1)	
Median (IQR)	295 (121–608)	206 (83–486)	154 (53–389)	190 (74–463)	<0.001
**Disease characteristics**					
**Asthma onset age, yrs**	N = 160	N = 1494	N = 1105	N = 2759	
Mean (SD)	30.8 (18.3)	30.0 (19.0)	29.4 (19.1)	29.8 (19.0)	0.418
<18 yrs, n (%)	43 (26.9)	454 (30.4)	345 (31.2)	842 (30.5)	0.649
≥18 yrs, n (%)	117 (73.1)	1040 (69.6)	760 (68.8)	1917 (69.5)	
**Asthma duration, yrs**	N = 160	N = 1470	N = 1081	N = 2711	
Mean (SD)	21.1 (15.2)	22.2 (16.4)	23.9 (16.9)	22.8 (16.5)	0.010
**Eosinophil phenotype**^b^	N = 176	N = 1936	N = 1586	N = 3698	
Non eosinophilic, n (%)	5 (2.8)	77 (4.0)	0 (0.0)	82 (2.2)	<0.001
Least likely, n (%)	12 (6.8)	117 (6.0)	0 (0.0)	129 (3.5)	
Likely, n (%)	7 (4.0)	69 (3.6)	126 (7.9)	202 (5.5)	
Most likely, n (%)	152 (86.4)	1673 (86.4)	1460 (92.1)	3285 (88.8)	
**Allergen tests** + ve SPT and/or + ve SAT, n (%)	N = 171 98 (57.3)	N = 1164 743 (63.8)	N = 561 301 (53.7)	N = 1896 1142 (60.2)	<0.001
**Exacerbations**	N = 215	N = 2327	N = 1488	N = 4030	
Mean (SD)	0 (0)	3.5 (4.1)	3.9 (4.8)	3.5 (4.4)	<0.001
0, n (%)	215 (100.0)	0 (0)	179 (12.0)	394 (9.8)	<0.001
1, n (%)	0 (0)	737 (31.7)	338 (22.7)	1075 (26.7)	
2, n (%)	0 (0)	498 (21.4)	235 (15.8)	733 (18.2)	
3, n (%)	0 (0)	317 (13.6)	161 (10.8)	478 (11.9)	
≥4, n (%)	0 (0)	775 (33.3)	575 (38.6)	1350 (33.5)	
**Asthma control**^c^	N = 175	N = 1521	N = 1117	N = 2813	
Well controlled, n (%)	48 (27.4)	147 (9.7)	106 (9.5)	301 (10.7)	0.849
Partly controlled, n (%)	58 (33.1)	254 (16.7)	178 (15.9)	490 (17.4)	
Un-controlled, n (%)	69 (39.4)	1120 (73.6)	833 (74.6)	2022 (71.9)	
**Lung function**^d^	N = 170	N = 1616	N = 917	N = 2703 74.8 (22.6) 1599 (59.2)	
ppFEV_1_, mean (SD)	80.9 (19.9)	75.2 (22.0)	72.8 (23.9)		0.011
ppFEV_1_ <80%, n (%)	87 (51.2)	942 (58.3)	570 (62.2)		0.057
	N = 168	N = 1627	N = 1389	N = 3184	
FEV_1_/FVC, median (IQR)	0.70 (0.61–0.78)	0.70 (0.61–0.77)	0.66 (0.56–0.75)	0.69 (0.59–0.76)	<0.001
FEV_1_/FVC <0.7, n (%)	83 (49.4)	805 (49.5)	812 (58.5)	1700 (53.4)	<0.001
**Asthma treatment**					
**Daily ICS dose,**^e^**μg**	N = 68	N = 972	N = 605	N = 1645	
Mean (SD)	540 (565)	578 (768)	511 (710)	552 (740)	
Median (IQR)	400 (52–1000)	370 (160–750)	320 (100–585)	360 (142–690)	0.004
>0–125 μg, n (%)	23 (33.8)	207 (21.3)	164 (27.1)	394 (23.9)	0.102
>125–250 μg, n (%)	7 (10.3)	179 (18.4)	110 (18.2)	296 (18.0)	
>250–500 μg, n (%)	12 (17.6)	241 (24.8)	143 (23.6)	396 (24.1)	
>500–1000 μg, n (%)	16 (23.5)	250 (25.7)	137 (22.6)	403 (24.5)	
>1000 μg, n (%)	10 (14.7)	95 (9.8)	51 (8.4)	156 (9.5)	
**LTOCS daily dose,**^f^**mg**			N = 1687		
Mean (SD)	–	–	12.2 (9.6)	–	
≤5 mg	–	–	463 (27.4)	–	
>5 mg to ≤10 mg	–	–	610 (36.2)	–	
>10 to ≤20 mg	–	–	395 (23.4)	–	
>20 mg	–	–	219 (13.0)	–	
**Add on to ICS/LABA**					
LAMA, n (%)	24 (11.2)	562 (24.1)	551 (31.3)	1137 (26.4)	<0.001
LTRA, n (%)	45 (20.9)	669 (28.7)	564 (32.0)	1278 (29.7)	0.021
Theophylline, n (%)	5 (2.3)	84 (3.6)	191 (10.9)	280 (6.5)	<0.001
Macrolide, n (%)	3 (1.4)	94 (4.0)	122 (6.9)	219 (5.1)	<0.001
**Total OCS dosage, mg**^f^		N = 2330	N = 1687	N = 4232	
Cumulative OCS dose last 90 days, mean (SD)	–	361.3 (864.2)	1522.6 (1964.6)	805.9 (1515.2)	<0.001
		N = 2294	N = 1687	N = 3981	
Total OCS last yr, mean (SD)	–	952.2 (2822.3)	5453.4 (7569.5)	2859.7 (5814.7)	<0.001
**Subsequent biologic**					
Anti-IgE, n (%)	83 (38.6)	766 (32.9)	426 (24.2)	1275 (29.6)	
Anti-IL5/5R, n (%)	110 (51.2)	1321 (56.7)	1215 (69.0)	2646 (61.5)	<0.001
Anti-IL4Rα, n (%)	22 (10.2)	240 (10.3)	116 (6.6)	378 (8.8)	
Anti-TSLP, n (%)	0 (0.0)	3 (0.1)	3 (0.2)	6 (0.1)	
**Healthcare resource utilisation**					
**Asthma related ED visits**	N = 186	N = 1860	N = 1475	N = 3521	
Mean (SD)	0 (0)	1 (3.1)	0.6 (2.4)	0.8 (2.8)	<0.001
0, n (%)	186 (100.0)	1338 (71.9)	1175 (79.7)	2699 (76.6)	<0.001
1, n (%)	0 (0.0)	203 (10.9)	116 (7.9)	319 (9.1)	
2+, n (%)	0 (0.0)	319 (17.2)	184 (12.5)	503 (14.3)	
**Asthma-related hospitalizations**	N = 186	N = 1886	N = 1476	N = 3548	
Mean (SD)	0 (0)	0.4 (1.0)	0.6 (1.7)	0.4 (1.3)	0.204
0, n (%)	186 (100.0)	1510 (80.1)	1164 (78.9)	2860 (80.6)	
1, n (%)	0 (0.0)	218 (11.6)	147 (10.0)	365 (10.3)	0.012
2+, n (%)	0 (0.0)	158 (8.4)	165 (11.2)	323 (9.1)	

BMI: body mass index; Cum: cumulative; ED: emergency department; ICS: inhaled corticosteroids; Ig: immunoglobulin; IL: interleukin; iOCS: intermittent oral corticosteroids; IQR: interquartile range; FEV_1_: forced expiratory volume in 1 s; FVC: forced vital capacity; LABA: long-acting β2-agonist; LAMA: long-acting muscarinic antagonist; LTRA: leukotriene receptor antagonist; LTOCS: long-term corticosteroids; ppFEV_1_: percent predicted forced expiratory volume in 1 s; SD: standard deviation; TSLP: thymic stromal lymphopoietin; SPT: skin prick test; SAT: serum allergen test.

a iOCS: ≤90 days in last 12 months; LTOCS >90 days in last 12 months.

b According to the expert consensus framework of Heaney et al., 2021^4^.

c Defined by GINA 2020 control test, asthma control questionnaire (ACQ) or asthma control test (ACT) (country specific). Conversion of ACT and ACQ to GINA control criteria as follows: ACQ - Mean ACQ ≤0.75 = Well controlled; 0.75 < Mean ACQ <1.5 = Partly controlled; Mean ACQ ≥1.5 = UncontrolledACT - Total ACT >19 = Well controlled; 15< Total ACT ≤19 = Partly controlled; Total ACT ≤15 = Uncontrolled.

d For FEV_1_, post-bronchodilator measures were used if available, and pre-bronchodilator measures otherwise, while ensuring that pre- and post-biologic measures were both either pre- or post-bronchodilator. In the sub-population of patients included in the lung function analysis (N = 3184), post-bronchodilator measurements were used for 68.8% patients.

e Beclomethasone equivalent.

f Prednisone equivalent

### Phenotypic characterization by OCS treatment pattern

Overall, 5.0% (n = 215), 54.1% (n = 2330) and 40.9% (n = 1760) of patients had no prescription for OCS, iOCS, and LTOCS, respectively (Table 1). OCS burden was high, with a cumulative OCS dose (prednisolone equivalent) in the last 90 days of 361 (SD 864) mg and 1523 (SD 1965) mg in the iOCS and LTOCS groups, respectively (p < 0.001). 36.4% of patients in the LTOCS group were prescribed a daily dose >10 mg and 46.9% of the iOCS group had a prescription for 3 or more courses/year.

### Biomarker distribution by OCS treatment pattern

**Fig. 2:**
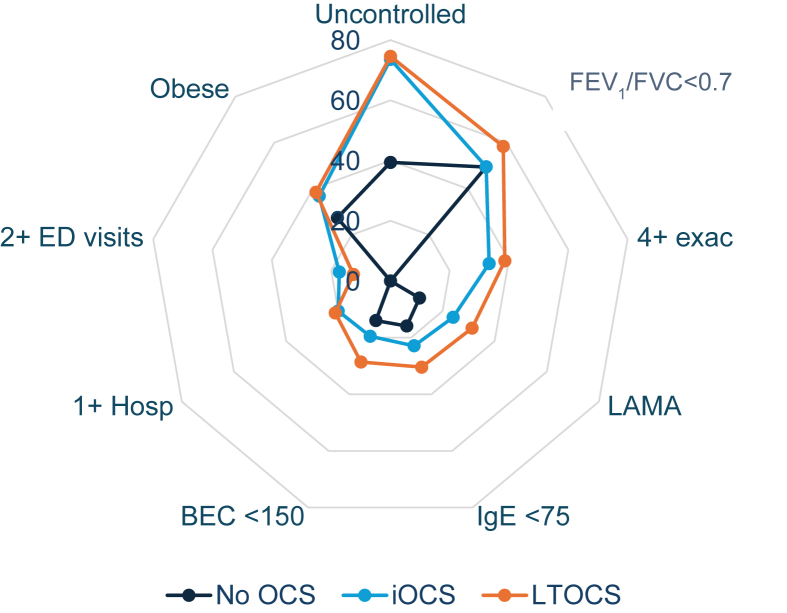
Severe asthma phenotypes by pattern of OCS use BEC: blood eosinophil count; ED: emergency department; exac: exacerbation; FEV_1_: forced expiratory volume in 1 s; FVC: forced vital capacity; IgE: immunoglobulin E; Hosp: asthma-related hospitalization; iOCS: intermittent oral corticosteroid; LAMA: long-acting muscarinic antagonist; LTOCS: long-term oral corticosteroid; OCS: oral corticosteroid; Intermittent: OCS use for ≤90 days; Long-term: OCS use for >90 days; Exacerbation: an asthma-related hospital attendance/admission and/or an asthma related emergency room attendance, and/or an acute OCS course of ≥3 days; Uncontrolled: assessed by GINA control criteria, ACT or ACQ (country specific) ACT and ACQ converted to GINA control criteria as outlined in Table 1 footer; Obese: BMI ≥30 kg/m^2^

OCS prescription was associated with variable changes in biomarker distribution, for both intermittent and long-term OCS groups (Fig. 1A-C). A leftward shift in BEC distribution, towards lower BEC, was observed in both iOCS and LTOCS groups relative to no OCS, but was more apparent in the LTOCS group, (standardized mean differences compared with no OCS were 0.07 and 0.14, respectively) suggesting a general reduction in BEC level (Fig. 1A). In total, 28.6% (n = 369/1288) of LTOCS patients had a BEC <150 cells/μL compared to 19.5% (n = 284/1460) of iOCS patients and 14.0% (n = 21/150) of those not treated with OCS. The median BEC was also significantly lower in the LTOCS group relative to the iOCS group (310 cells/μL vs. 400; p < 0.001) (Table 1). This BEC distribution leftward shift occurred even at a LTOCS daily dose of ≤5 mg but was more apparent at higher doses (S-Fig. 2). However, some patients (59%) still had a high BEC (≥300 cell/μL) despite LTOCS prescription; >90% of these patients had an LTOCS prescription at the time of latest BEC measurement. A similar trend was noted for total serum IgE: 30.4% (n = 237/778) of LTOCS patients had an IgE <75 IU/mL compared to 22.9% (n = 291/1272) of iOCS patients and 15.9% (n = 23/145) of non-OCS patients (Fig. 1C). Notably, a similar trend was noted when excluding those patients with a positive skin prick test or serum specific allergen test, thereby accounting for differences in atopy between groups (S-Table 5). The median IgE concentration was significantly lower in those prescribed LTOCS (154 IU) compared to those prescribed iOCS (206 IU; p < 0.001) (Table 1). The impact of OCS was less apparent for FeNO, where a similar proportion of patients had an FeNO <25 ppb–38.9%, 37.5%, and 31.9% in the no OCS, iOCS, and LTOCS groups, respectively (Fig. 1B). However, the median FeNO level was significantly higher in LTOCS vs. iOCS group (40 vs 34 ppb; p < 0.001) (Table 1), and a greater proportion of LTOCS than iOCS patients retained a high FeNO (≥25 ppb) in the presence of low BEC (<300 cells/μL; 27.0 vs 16.7%; S-Table 6).

### Clinical characteristics and healthcare resource utilisation by OCS prescription pattern

Unadjusted comparisons showed that patients receiving OCS, either long-term or intermittently, were also more likely to be obese, have uncontrolled asthma and impaired FEV_1_, have experienced ≥4 exacerbations, have received long-acting muscarinic antagonist (LAMA) add-on therapy, and have been hospitalized or visited the ED for their asthma in the last year, than their non-OCS counterparts (Table 1; Fig. 2). A greater proportion of LTOCS than iOCS patients had irreversible airflow obstruction (58.5 vs 49.5%; p < 0.001), ≥4 exacerbations (38.6 vs 33.3%; p < 0.001), and add-on therapies to ICS/LABA, (Table 1; Fig. 2). Conversely, a greater proportion of iOCS than LTOCS patients had a positive skin prick and/or serum allergen test (63.8 vs 53.7%; p < 0.001). Phenotypic characteristics were broadly similar across LTOCS doses (S-Table 7), with the exception of exacerbation frequency and HCRU which was slightly higher in those in the highest LTOCS daily dose category (>20 mg) vs. the other dose categories (i.e. ≤5 mg, >5 to ≤10 mg, and >10 to ≤20 mg).

### Disease characterization of LTOCS users by BEC cut-off

**Fig. 3:**
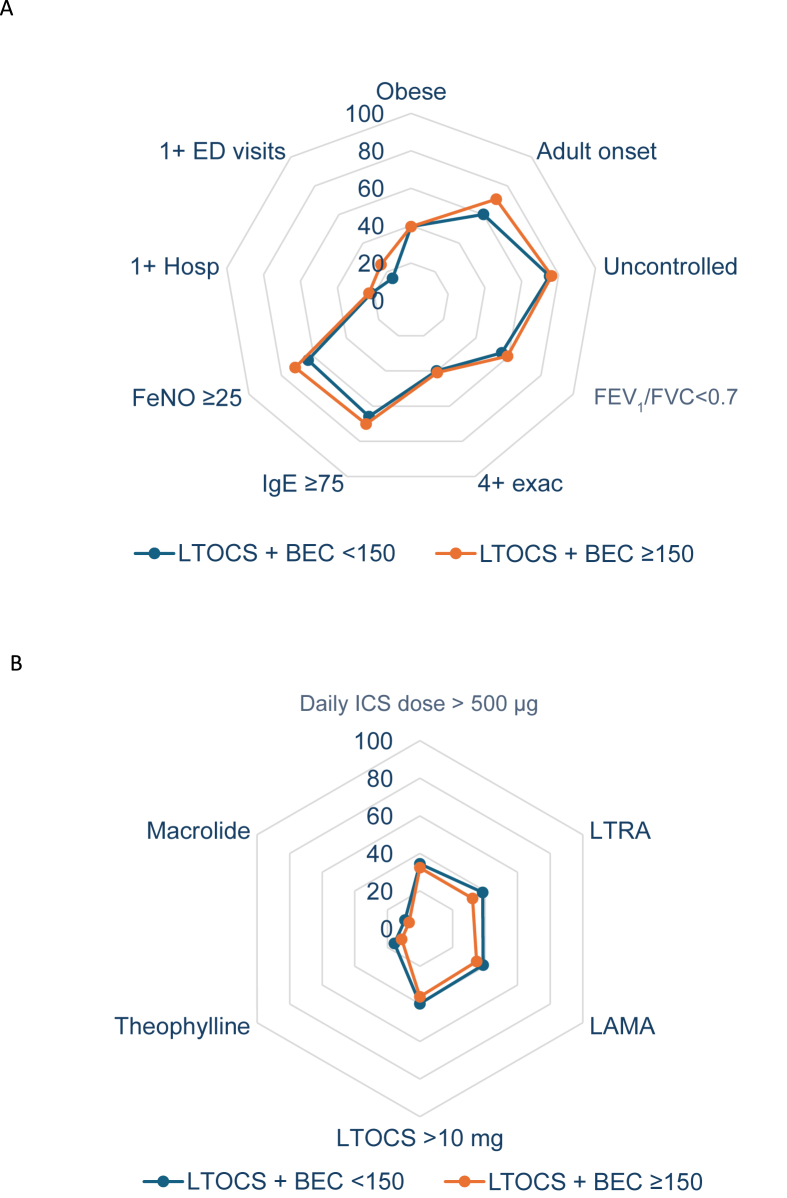
Disease characteristics of patients with severe asthma treated with LTOCS by BEC cut-off BEC: blood eosinophil count; ED: asthma related emergency department visit; exac: exacerbation; FeNO: fractional exhaled nitric oxide; FEV_1_: forced expiratory volume in 1 s; FVC: forced vital capacity; Hosp: asthma-related hospitalization; IgE: immunoglobulin E; LTOCS: long-term oral corticosteroid (>90 days) Obese: >30 kg/m^2^; Adult onset: onset at ≥18 years; Exacerbation: an asthma-related hospital attendance/admission and/or an asthma related emergency room attendance, and/or an acute OCS course of ≥3 days; Uncontrolled: assessed by GINA control criteria, ACT or ACQ (country specific) ACT and ACQ converted to GINA control criteria as outlined in Table 1 footer; ICS dose: BDP equivalent

A total of 1288 LTOCS patients had pre-biologic BEC data; 28.6% (n = 369) had a BEC <150 cells/μL and 71.4% (n = 919) had a BEC ≥150 cells/μL (Table 2). Demographic characteristics were similar between groups (Table 2; Fig. 3). Broadly similar IgE profiles were noted for both BEC cohorts, but more patients with FeNO <25 ppb were noted in the low BEC cohort (36.4% vs 28.5%; p = 0.028) (Table 2; Fig. 3). Those in the low BEC group tended to be younger at asthma onset (26.5 vs 29.9 yrs; p = 0.021) and have a longer asthma duration (26.0 vs 23.2 yrs; p = 0.036) (Table 2).

**Table 2:** Demographic and clinical characteristics plus HCRU pre-biologic initiation for severe asthma patients treated with LTOCS, stratified by BEC cut-off.

	LTOCS^a^ + BEC <150 cells/μL (N = 369)	LTOCS^a^ + BEC ≥150 cells/μL (N = 919)	P-value
Demographic characteristics			
**Sex**			
Female, n (%)	225 (61.0)	555 (60.4)	0.846
**Age at biologic initiation, yrs**			
Mean (SD)	52.6 (13.8)	52.9 (14.2)	0.710
18-34, n (%)	43 (11.7)	124 (13.5)	0.689
35-54, n (%)	143 (38.8)	340 (37.0)	
55-79, n (%)	180 (48.8)	443 (48.2)	
≥80, n (%)	3 (0.8)	12 (1.3)	
**BMI, Kg/m^2^**	N = 331	N = 810	
Underweight, n (%)	8 (2.4)	21 (2.6)	0.996
Normal, n (%)	93 (28.1)	231 (28.5)	
Overweight, n (%)	99 (29.9)	238 (29.4)	
Obese (≥30), n (%)	131 (39.6)	320 (39.5)	
**Smoking status**	N = 306	N = 778	
Current smoker, n (%)	9 (2.9)	14 (1.8)	0.052
Ex-smoker, n (%)	83 (27.1)	266 (34.2)	
Never smoked, n (%)	214 (69.9)	498 (64.0)	
Biomarkers			
**FeNO (ppb)**	N = 236	N = 520	
Mean (SD)	57.0 (54.3)	59.9 (54.6)	
Median (IQR)	38 (19–74)	41 (22–78)	0.241
<25 ppb, n (%)	86 (36.4)	148 (28.5)	0.028
≥25 ppb, n (%)	150 (63.6)	372 (71.5)	
**IgE, IU/mL**	N = 173	N = 443	
Mean (SD)	471.2 (1209.5)	353.9 (646.3)	
Median (IQR)	129 (42–370)	158 (56–389)	0.409
<75 IU/mL, n (%)	59 (34.1)	132 (29.8)	0.299
≥75 IU/mL, n (%)	114 (65.9)	311 (70.2)	
Disease characteristics			
**Asthma onset age, yrs**	N = 218	N = 632	
Mean (SD)	26.5 (20.0)	29.9 (18.6)	0.021
<18 yrs, n (%)	87 (39.9)	185 (29.3)	0.004
≥18 yrs, n (%)	131 (60.1)	447 (70.7)	
**Asthma duration, yrs**	N = 213	N = 615	
Mean (SD)	26.0 (17.6)	23.2 (16.2)	0.036
**Eosinophil phenotype**^b^	0 (0.0)	0 (0.0)	
Non eosinophilic, n (%)	0 (0.0)	0 (0.0)	<0.001
Least likely, n (%)	126 (34.1)	0 (0.0)	
Likely, n (%)	243 (65.9)	919 (100.0)	
Most likely, n (%)			
**Allergen tests**			
+ve SPT, n(%)	N = 46	N = 231	
	30 (65.2)	109 (47.2)	0.026
+ve SAT, n(%)	N = 24	N = 81	
	14 (58.3)	50 (61.7)	0.765
+ve SPT and/or + ve SAT, n (%)	N = 64	N = 275	
	39 (60.9%)	137 (49.8%)	0.109
**Exacerbations**	N = 298	N = 822	
Mean (SD)	4.0 (5.5)	4.2 (4.5)	0.135
0, n (%)	33 (11.1)	78 (9.5)	0.834
1, n (%)	66 (22.1)	179 (21.8)	
2, n (%)	43 (14.4)	136 (16.5)	
3, n (%)	37 (12.4)	93 (11.3)	
≥4, n (%)	119 (39.9)	336 (40.9)	
**Asthma control**^c^	N = 221	N = 604	
Well controlled, n (%)	29 (13.1)	44 (7.3)	0.013
Partly controlled, n (%)	26 (11.8)	100 (16.6)	
Uncontrolled, n (%)	166 (75.1)	460 (76.2)	
**Lung function**^d^	N = 158	N = 445	
ppFEV_1_, mean (SD)	71.4 (23.5)	72.8 (24.2)	0.519
ppFEV_1_ <80%, n (%)	109 (69.0)	271 (60.9)	0.070
	N = 306	N = 776	
FEV_1_/FVC, median (IQR)	0.67 (0.57–0.76)	0.66 (0.56–0.74)	0.221
FEV_1_/FVC <0.7, n (%)	171 (55.9)	462 (59.5)	0.272
Asthma treatment			
**Daily ICS dose,**^e^**μg**	N = 139	N = 299	
Mean (SD)	608 (925)	518 (689)	
Median (IQR)	400 (160–610)	320 (100–640)	0.192
>0–125 μg, n (%)	31 (22.3)	84 (28.1)	0.234
>125–250 μg, n (%)	19 (13.7)	55 (18.4)	
>250–500 μg, n (%)	41 (29.5)	63 (21.1)	
>500–1000 μg, n (%)	36 (25.9)	70 (23.4)	
>1000 μg, n (%)	12 (8.6)	27 (9.0)	
**LTOCS daily dose,**^f^**mg**	N = 353	N = 883	
Mean (SD)	12.3 (9.1)	12.4 (9.7)	0.308
≤5 mg	76 (21.5)	250 (28.3)	0.001
>5 mg to ≤10 mg	136 (38.5)	313 (35.4)	
>10 to ≤20 mg	108 (30.6)	199 (22.5)	
>20 mg	33 (9.3)	121 (13.7)	
**Add on to ICS/LABA**			
LAMA, n (%)	143 (38.8)	320 (34.8)	0.184
LTRA, n (%)	142 (38.5)	297 (32.3)	0.035
Theophylline, n (%)	58 (15.7)	103 (11.2)	0.027
Macrolide, n (%) (SD)	34 (9.2)	62 (6.7)	0.127
**Total OCS dosage**^f^			
Cumulative OCS dose last 90 days (mg), mean (SD)	1805.7 (2286.6)	1517.3 (1965.6)	0.005
Total OCS last yr (mg), mean (SD)	6421.7 (8889.8)	5300.5 (7396.9)	0.006
**Subsequent biologic**			
Anti-IgE, n (%)	122 (33.1)	174 (18.9)	<0.001
Anti-IL5/5R, n (%)	226 (61.2)	691 (75.2)	
Anti-IL4Rα, n (%)	18 (4.9)	54 (5.9)	
Anti-TSLP, n (%)	3 (0.8)	0 (0.0)	
Healthcare resource utilisation			
**Asthma-related ED visits**	N = 309	N = 777	<0.001
Mean (SD)	0.3 (1.0)	0.9 (3.1)	
0, n (%)	261 (84.5)	582 (74.9)	0.002
1, n (%)	23 (7.4)	74 (9.5)	
2+, n (%)	25 (8.1)	121 (15.6)	
**Asthma-related hospitalizations**	N = 309	N = 773	
Mean (SD)	0.5 (1.2)	0.7 (1.8)	0.497
0, n (%)	242 (78.3)	597 (77.2)	0.110
1, n (%)	37 (12.0)	71 (9.2)	
2+, n (%)	30 (9.7)	105 (13.6)	

BMI: body mass index; ED: emergency department; ICS: inhaled corticosteroids; Ig: immunoglobulin; IL: interleukin; iOCS: intermittent oral corticosteroids; IQR: interquartile range; FEV_1_: forced expiratory volume in 1 s; FVC: forced vital capacity; LABA: long-acting β2-agonist; LAMA: long-acting muscarinic antagonist; LTRA: leukotriene receptor antagonist; LTOCS: long-term corticosteroids; ppFEV_1_: percent predicted forced expiratory volume in 1 s; SD: standard deviation; TSLP: thymic stromal lymphopoietin; SPT: skin prick test; SAT: serum allergen test.

a LTOCS >90 days in last 12 months.

b According to the expert consensus framework of Heaney et al., 2021^4^.

c Defined by GINA 2020 control test, asthma control questionnaire (ACQ) or asthma control test (ACT) (country specific). Conversion of ACT and ACQ to GINA control criteria as follows:ACQ - Mean ACQ ≤0.75 = Well controlled; 0.75 < Mean ACQ <1.5 = Partly controlled; Mean ACQ ≥1.5 = UncontrolledACT - Total ACT >19 = Well controlled; 15< Total ACT ≤19 = Partly controlled; Total ACT ≤15 = Uncontrolled.

d For FEV_1_, post-bronchodilator measures were used if available, and pre-bronchodilator measures otherwise, while ensuring that pre- and post-biologic measures were both either pre- or post-bronchodilator. In the sub-population of patients included in the lung function analysis (N = 1082), post-bronchodilator measurements were used for 70.9% patients.

e Beclomethasone equivalent.

f Prednisone equivalent

LTOCS users with low BEC (<150 cells/μL) had a similar burden of disease compared to those with higher BEC (≥150 cells/μL) (Table 2; Fig. 3). Approximately 40% and 75% of patients experienced ≥4 exacerbations in the last year and had uncontrolled asthma symptoms respectively, irrespective of their BEC, although those with BEC <150 cells/μL were slightly more likely to have a ppFEV_1_ <80% (69.0% vs 60.9%). HCRU of LTOCS patients was largely independent of BEC, with a tendency for more asthma-related ED visits in the last year in the high vs. low BEC cohort (0.9 vs 0.3) (Table 2; Fig. 3).

Daily ICS dose, LTOCS daily dose, and add-on treatment to ICS/LABA was largely similar between groups, although cumulative and total OCS dose was slightly higher in the low BEC cohort (Table 2).

## Discussion

Our results are important as they describe the disease burden and biomarker distribution associated with OCS prescription in a large SA cohort in real-life by OCS prescription pattern. Categorization by LTOCS dose provided additional insight into the effect of OCS on biomarker distributions, and the OCS-sensitivity of biomarker levels. A leftward shift in BEC distribution was noted for patients prescribed LTOCS at a dose as low as <5 mg/day, which may mask T2-disease via down-regulation of genes involved in T2-inflammation (eg, IL4, IL5), leading to inappropriate treatment. Furthermore, we found that the burden of disease experienced by patients with SA prescribed LTOCS was independent of OCS dose or BEC. These patients may not qualify for biologic treatment in many countries emphasizing the urgent need to (i) develop alternative treatments for this subset of patients (representing 29% of the LTOCS cohort), (ii) consider phenotyping earlier in the asthma management journey or use of alternate methodology to phenotype more precisely (e.g. induced sputum, omics, etc.) and/or (iii) use alternative biologic eligibility BEC cut-offs for those prescribed OCS, either intermittently or long-term (as already applied in some countries).

Despite GINA's recommendation of using LTOCS as a last resort,^3^ approximately half of patients with SA included in our study were prescribed iOCS in the last year, and just over 40% of patients were prescribed LTOCS. Only 5% of patients had not received an OCS in the previous year, although this is perhaps unsurprising since OCS use is a requirement for biologic prescription in many countries.^26^ This is in keeping with data from a systematic review of studies from Europe, North America, and Asia which reported annual systemic or OCS prescription in approximately 50% of patients, with short-term use reported in up to 36% of patients.^12^ Dose and frequency of OCS use in our cohort were also high. This is clilnically relevant considering that the risk of OCS-reatled adverse events increases with higher daily systemic corticosteroid exposure and frequency of use,^15^, ^39^ that this association is relative consistent across age categories, GINA treatment steps and prior medication use,^18^ and the HCRU implications assoicated with managing OCS-related adverse events. Disease burden was also greater in those prescribed OCS, with these patients shown to have uncontrolled disease and irreversible airflow obstruction, irrespective of OCS prescription pattern and dose. Our findings, thus reaffirm GINA's position on sparing use of OCS and the need to carefully consider the risk to benefit ratio of OCS prescription, even for intermittent use. Whether earlier intervention with steroid-sparing treatments (e.g. biologics) in those with less severe disease, prior to significant lung function deterioration and OCS prescription, could be used as an alternative treatment strategy is the topic of several prospective trials currently underway.

OCS dose and frequency are also important to consider when conducting biomarker profiling. We found that OCS prescription was associated with a change in biomarker distribution pattern, relative to those with zero prescriptions, with a leftward shift noted for both BEC and IgE, irrespective of OCS prescription pattern, and at both high (>20 mg/day) and low (<5 mg/day) LTOCS dose, suggestive of a relatively flat dose-response relationship. In the absence of pre- and post-OCS data, the low BEC levels noted in those prescribed OCS in the current study could also be that these patients have innately low BEC, are steroid unresponsive, but receive OCS in ever increasing amounts by clinicians, who do not recognize the steroid non-responsiveness. However, others have reported a similar BEC lowering effect of OCS.^20^, ^21^, ^22^ For example, a meta-analysis including 61 studies found that OCS use reduced BEC by 76%, with an even greater reduction noted in corticosteroid-naïve patients (93%).^22^ From the opposite perspective, a *post-hoc* analysis of the SIRIUS study estimated that reduction of daily OCS dose by 5 mg/day led to a 41% increase in BEC.^40^ Importantly, BEC suppression has been shown to last for >24 h after a single OCS dose, and has also been reported with ICS (albeit to a lesser degree),^21^ not in itself surprising as the main action of steroids is against eosinophils. More surprisingly, however, not all patients with SA who were prescribed OCS in the current study, exhibited a low BEC. In fact, 58.6% and 66.3% of patients retained a high BEC (≥300 cells/μL) despite long-term and intermittent OCS prescription, respectively. This finding could be due to corticosteroid insensitivity,^41^ poor compliance with OCS,^42^ or a consequence of the high proportion of patients in our study with adult-onset disease (approx. 70%). Data from a Belgian SA cohort found a significant association between persistently high BEC and late-onset asthma in OCS-treated patients.^43^ Leftward shift changes in IgE concentration distribution in both OCS cohorts in the current study, with significantly lower concentrations observed in the LTOCS relative to the iOCS group, was a novel finding since previous research has suggested that corticosteroids slightly increase IgE concentrations, at least in the short term.^44^ However, the absence of pre-OCS data precludes an assessment of the magnitude of change. Future study assessing the impact (if any) of OCS on specific IgE concentrations is warranted.

We found less evidence of an OCS-masking effect for FeNO, suggesting that FeNO may be a more reliable biomarker to detect pulmonary T2-inflammation among OCS users. This finding may be due to differences in OCS sensitivity for systemic (ie, BEC) vs locally-produced (ie, FeNO) biomarkers. Indeed, FeNO has been reported to increase after systemic OCS treatment in some studies, likely due to its release following OCS-induced reduction in distal airway obstruction. However, others have reported a reduction in FeNO with OCS, with the vast majority of studies showing a FeNO reduction in the range 28–48%.^22^ FeNO non-suppression under high-dose treatment could, therefore, be useful in identifying corticosteroid non-responsiveness and in prompting consideration of biologic initiation.^45^ However, it is worth noting though that mean FeNO levels remained elevated (>30 ppb) post-OCS in 3 studies with high baseline FeNO (>50 ppb).^22^ The picture is further complicated by the fact that FeNO is affected both positively and negatively by non-asthma factors with elevation noted in patients with allergic rhinitis, but decreased in smokers with asthma or chronic obstructive pulmonary disease and in patients treated with high-dose ICS.^46^^,^^47^ However, we did find significantly higher FeNO in those prescribed OCS long-term rather than intermittently, which may represent residual T2-inflammation in the former group, or a consequence of higher ICS daily dose in the latter group.

Our results, and those of others, emphasize that biomarker profiling should not be conducted in isolation, as well as the importance of longitudinal biomarker assessment.^48^ For example, using a data-driven approach to cluster patients according to their BEC, FeNO and IgE concentrations along a gradient of T2-inflammatory biomarkers,16% of patients with SA had triple biomarker-low asthma.^34^ However, the proportion was much lower (1.6%), when considering BEC and FeNO cut-offs (highest ever value) in the context of LTOCS use, in addition to other clinical indices, including onset of asthma and presence of nasal polyps in an eosinophilic phenotype gradient algorithm, agreed by expert consensus.^4^ Specifically, among LTOCS users, an eosinophilic phenotype was graded as "most likely" (Grade 3) for those with BEC ≥150 to <300 cells/μL, and still considered "likely" (Grade 2) for those with BEC <150 cells/μL.^4^ Perhaps these, and other algorithms, should be updated to include iOCS use, frequency of use and biomarker cut-offs stratified by OCS dose, since mis-classification of endotype as T2-low has important implications for subsequent management. The practical challenge is to distinguish "pseudo-T2 low" patients due to LTOCS suppression of biomarkers from those with "true" T2-low disease.

Although treatment options in patients with low BEC in the presence of OCS may be limited, disease burden remains high and represents a significant unmet need in SA management. Patients in the LTOCS group with low BEC in the current study were just as likely as those with higher BEC to have uncontrolled disease, evidence of irreversible airflow obstruction and experience multiple exacerbations. This finding has been reported by others.^29^, ^30^, ^31^^,^^49^ Post-hoc analysis of the NOVELTY study, for example, found that 73% of patients with severe uncontrolled asthma had low BEC.^29^ Another US claims database study found that nearly 20% of patients with SA and low BEC, who had not been treated with biologics, also experienced exacerbations and had less than optimal asthma control in the 12 months following their most recent BEC measurement. Notably, maintenance systemic corticosteroid was observed in 22% of patients with a BEC of less than 300 cells/μL and 27% of patients with a BEC of less than 150 cells/μL.^49^ Data from Japan has yielded similar results.^31^ Patients receiving high-dose ICS and additional controller medications or OCS with evidence of T2-low disease (i.e. low BEC and low IgE) exhibited similar exacerbation rates and annual drug costs as those with T2-high disease.^31^

Limitations of our study include those common to all observational studies including referral bias and missing data; 33% and 51% of patients had missing BEC and FeNO data, respectively, reflective of inter-country differences in biomarker data collection practices. Only patients who subsequently received biologics were included and biomarker data were not collected pre-OCS and so we were unable to determine magnitude of change. Multiple biomarker measurements (rather than latest pre-biologic) and longitudinal assessment of biomarker concentrations would have provided a more granular look at the relationship between OCS use and biomarker distribution. Additionally, our definition of iOCS was broad (i.e. <90 days in last 12 months), incorporating a wide variation of OCS use and could have led to misclassification of OCS prescription pattern. The exact temporal relationship between OCS use and the testing of BEC was not available. However, in the current study OCS were prescribed intermittently as short courses of rescue steroids (≥3 days/course) for the treatment of exacerbations in the majority of cases; 46.9% of the iOCS group had a prescription for 3 or more courses/year. OCS use did not include intravenous or intramuscular administration. Strengths include inclusion of a large heterogenous, real-life, SA population, encompassing patients from 23 countries and multiple centres, and representative of the general SA population. Assessment of the impact of OCS use on 3 commonly used biomarkers in severe asthma, namely BEC, FeNO, and IgE, is critical for understanding asthma phenotypes and tailoring treatment strategies, especially in the context of biologic therapy. Furthermore, collection of a wide range of demographic and clinical variables also facilitated comprehensive phenotypic characterization, allowing for a nuanced analysis of how different patterns of corticosteroid use affect asthma characteristics and treatment outcomes. The use of robust statistical analyses to compare cohorts adds credibility to our findings. Further work is needed to investigate the temporal relationship of OCS use and biomarker concentrations (eg, different seasons and time of day), in alternate populations (eg, paediatrics) and assessed by country, to assess the impact (if any) of OCS adherence on biomarker distribution, and to describe phenotypes of LTOCS patients using alternative biomarkers (eg, FeNO, specific IgE), cut-offs and combinations.

In conclusion, OCS, whether prescribed intermittently or long-term, affects BEC distribution, which may compromise T2-stratification and subsequent treatment decisions, but disease burden remains high among LTOCS users irrespective of BEC. FeNO concentrations were relatively preserved suggesting its utility for endotyping in an environment of LTOCS use, and its potential to more reliability identify those, who may benefit from a T2-targeted biologic. Our findings emphasize the importance of taking into account OCS use, even intermittent use, when endotyping SA, and suggest the need for endotyping and identification of pertinent biomarkers earlier in the asthma management pathway, prior to initiation of LTOCS (early disease phenotyping). Moreover, there is a need to find alternative treatment strategies for LTOCS users with low BEC, who represent about 1/4 of SA patients. Importantly, there is a significant unmet need among patients with SA treated with OCS (either intermittently or long-term) with low BEC, who may not qualify for most biologic therapies.

## Abbreviations

ACQ, asthma control questionnaire; ACT, asthma control test; ADEPT, Anonymized Data Ethics Protocols and Transparency Committee; BEC, blood eosinophil count; BMI, body mass index; ED, emergency department; ENCePP, European Network Centres for Pharmacoepidemiology and Pharmacovigilance; exac, exacerbation; FeNO, fractional exhaled nitric oxide; FEV_1_, forced expiratory volume in 1 s; FVC, forced vital capacity; GINA, Global Initiative for Asthma; HCRU, healthcare resource utilisation; Hosp, asthma-related hospitalization; ICS, inhaled corticosteroids; Ig, immunoglobulin; IgE, immunoglobulin E; IL, interleukin; iOCS, intermittent oral corticosteroid; IQR, interquartile range; ISAR, International Severe Asthma Registry; LABA, long-acting β2-agonist; LAMA, long-acting muscarinic antagonist; LTOCS, long-term oral corticosteroid; LTRA, leukotriene receptor antagonist; OCS, oral corticosteroid; ppFEV1, percent predicted forced expiratory volume in 1 s; SA, severe asthma; SAT, serum allergen test; SD, standard deviations; SPT, skin prick test; TSLP, thymic stromal lymphopopoietin.

## Data sharing

The dataset supporting the conclusions of this article was derived from the International Severe Asthma Registry (ISAR). This study was approved by the Anonymized Data Ethics Protocols and Transparency (ADEPT) committee – the independent scientific advisory committee for ISAR. The authors do not have permission to give public access to the study dataset; researchers may request access to ISAR data for their own purposes. ISAR research requests and proposals can be made via the ISAR website (https://isaregistries.org/research-proposal-requests/) or via the enquiries email to info@isaregistries.org. In line with ISAR governance restrictions, sharing individual deidentified participant data is subject to the consent of the ISAR steering committee in accordance with patient consent, patient confidentiality and ethical considerations. The study documents (protocol, statistical analysis plan, clinical study report) will be made available in accordance with the criteria of the European Network of Centres for Pharmacoepidemiology and Pharmacovigilance (EUPAS49201). Proposals should be directed to info@isaregistries.org; to gain access, if approved by the regulatory boards, data requestors will need to sign a data access agreement.

## Author contributions

All authors contributed to the design of the work, analysis and interpretation of data, drafting of the manuscript and manuscript revisions.

All authors contributed to interpretation of data, and revising the article critically for important intellectual content and clinical relevancy.

All authors have approved the final version to be published and to be accountable for all aspects of the work in ensuring that questions related to the accuracy or integrity of any part of the work are appropriately investigated and resolved.

## Ethics statement

This study was designed, implemented, and reported in compliance with the European Network Centres for Pharmacoepidemiology and Pharmacovigilance Code of Conduct (EMA 2014; EUPAS49201) and with all applicable local and international laws and regulation. Registration of the ISAR database with the European Union Electronic Register of Post-Authorization studies was also undertaken (ENCEPP/DSPP/23720). ISAR has ethical approval from the Anonymized Data Ethics Protocols and Transparency (ADEPT) committee (ADEPT0218). Governance was provided by The Anonymous Data Ethics Protocols and Transparency (ADEPT) committee (registration number: ADEPT1022). All data collection sites in the International Severe Asthma Registry (ISAR) have obtained regulatory agreement in compliance with specific data transfer laws, country-specific legislation, and relevant ethical boards and organizations.

## Submission declaration

The authors confirm that the manuscript is original, has not been published before, and is not currently being considered for publication elsewhere.

## Funding statement

This study was conducted by the Observational and Pragmatic Research Institute (OPRI) Pte Ltd and was partially funded by Optimum Patient Care Global (OPCG), Sanofi and Regeneron Pharmaceuticals, Inc. The International Severe Asthma Registry (ISAR) is operated by OPCG and co-funded by OPCG and AstraZeneca. OPCG paid for medical writing support.

## Declaration of competing interest

**Florence Schleich** reports consultancy work for GlaxoSmithKline, AstraZeneca, Sanofi - Advisory board, received speaker fees from GlaxoSmithKline, AstraZeneca, Chiesi, Teva, ALK and research grants from GlaxoSmithKline, AstraZeneca, and Chiesi.

**Désirée Larenas-Linnemann** reports personal fees from ALK-Abelló, AstraZeneca national and global, Bayer, Chiesi, Grunenthal, Grin, GlaxoSmithKline national and global, Viatris, Novartis, Pfizer, Sanofi, Siegfried, and Carnot, grants for guideline development from Abbvie, Bayer, Chiesi, Lilly, Sanofi, AstraZeneca, Pfizer, Novartis, Circassia, UCB, and GlaxoSmithKline, outside the submitted work.

**Alan Altraja** has received lecture fees from AstraZeneca, Berlin-Chemie Menarini, Boehringer Ingelheim, CSL Behring, GlaxoSmithKline, Merck, Sharp & Dohme, Norameda, Novartis, Orion, Sanofi, Takeda, Teva, and Zentiva; sponsorships from AstraZeneca, Berlin-Chemie Menarini, Boehringer Ingelheim, CSL Behring, GlaxoSmithKline, Merck, Sharp & Dohme, Norameda, Novartis, Takeda, Teva, and Sanofi; and has participated in advisory boards for AstraZeneca, Boehringer Ingelheim, CSL Behring, GlaxoSmithKline, Merck, Sharp & Dohme, Novartis, Sanofi, Shire Pharmaceuticals, and Teva.

**Luis PérezdeLlano** reports grants, personal fees, and non-financial support from AstraZeneca, personal fees and non-financial support from GlaxoSmithKline, grants, personal fees and non-financial support from Teva, personal fees and non-financial support from Chiesi, grants, personal fees and non-financial support from Sanofi, personal fees from Merck Sharp & Dohme, personal fees from Techdow Pharma, grants, personal fees and non-financial support from Faes Farma, personal fees from Leo-Pharma, grants and personal fees from Gebro, personal fees from Gilead, outside the submitted work.

**Konstantinos Kostikas** received honoraria for presentations and consultancy fees from AstraZeneca, Boehringer Ingelheim, Chiesi, ELPEN, GlaxoSmithKline, Guidotti, Menarini, Pfizer, Sanofi, Specialty Therapeutics. He was an employee of AstraZeneca from 2 September to 29 November2024.

**Mohsen Sadatsafavi** has received honoraria from AstraZeneca, Boehringer Ingelheim, Teva, and GlaxoSmithKline for purposes unrelated to the content of this manuscript and has received research funding from AstraZeneca and Boehringer Ingelheim directly into his research account from AstraZeneca for unrelated projects.

**Arnaud Bourdin** has received industry-sponsored grants from AstraZeneca/MedImmune, Boehringer Ingelheim, Cephalon/Teva, GlaxoSmithKline, Novartis, Sanofi-Regeneron, and consultancies with AstraZeneca/MedImmune, Boehringer Ingelheim, GlaxoSmithKline, Novartis, Regeneron-Sanofi, Med-in-Cell, Actelion, Merck, Roche, and Chiesi.

**Roy Alton Pleasants** is a consultant for AstraZeneca and Grifols and receives research support through institutions from AstraZeneca and GlaxoSmithKline.

**Mark Hew** declares grants and other advisory board fees (made to his institutional employer) from AstraZeneca, GlaxoSmithKline, Novartis, Sanofi, Teva, and Seqirus, for unrelated projects.

**Wenjia Chen** reports no conflict of interest.

**Libardo Jiménez-Maldonado** has received fees as advisory board participant and/or speaker from AstraZeneca, Boehringer-Ingelheim, GlaxoSmithKline, Novartis, and Sanofi-Aventis; has participated in clinical trials for AstraZeneca, Novartis and GlaxoSmithKline.

**Simon Couillard** reports the following: he has received non-restricted research grants from the NIHR Oxford BRC, the Quebec Respiratory Health Research Network, the Fondation Québécoise en Santé Respiratoire, AstraZeneca, bioMérieux, and Sanofi-Genyme-Regeneron; he is the holder of the Association Pulmonaire du Québec's Research Chair in Respiratory medicine and is a Clinical research scholar of the Fonds de recherche du Québec; he received speaker honoraria from AstraZeneca, GlaxoSmithKline, Sanofi-Regeneron, and Valeo Pharma; he received consultancy fees for FirstThought, AstraZeneca, GlaxoSmithKline, Sanofi-Regeneron, Access Biotechnology and Access Industries; he has received sponsorship to attend/speak at international scientific meetings by/for AstraZeneca and Sanofi-Regeneron. He is an advisory board member and will have stock options for Biometry Inc – a company which is developing a FeNO device (myBiometry). He advised the Institut national d'excellence en santé et services sociaux (INESSS) for an update of the asthma general practice information booklet for general practitioners.

**Charlotte Suppli Ulrik** reports personal fees for talks, participation in advisory boards etc. from AstraZeneca, GlaxoSmithKline, Teva, Boehringer Ingelheim, Orion Pharma, Sanofi Genzyme, TFF Pharmaceuticals, Covis Pharma, Berlin-Chemie, Takeda, Chiesi, Pfizer, Hikma Pharmaceuticals, and Novo Nordisk, outside the submitted work.

**Adeeb A. Bulkhi** has received speaker lecture fees from AstraZeneca, GlaxoSmithKline, Sanofi, Novartis, Takeda, and ALK. He also participated in advisory boards with GlaxoSmithKline, Sanofi, and Novartis.

**Ming-Ju Tsai** has received sponsorship to attend or speak at conferences, honoraria for lecturing or attending advisory boards, and research grants from the following companies: AstraZeneca, Boehringer Ingelheim, GlaxoSmithKline, Novartis, Pfizer, Shionogi and Orient EuroPharma.

**George C. Christoff** declares relevant research support from AstraZeneca and Sanofi.

**Nikolaos G. Papadopoulos** has been a speaker and/or advisory board member for Abbott, Abbvie, ALK, Asit Biotech, AstraZeneca, Biomay, Boehringer Ingelheim, GlaxoSmithKline, HAL, Faes Farma, Medscape, Menarini, Merck Sharp & Dohme, Novartis, Nutricia, OM Pharma, Regeneron, Sanofi, Takeda, and Viatris.

**Paul E. Pfeffer** has attended advisory boards for AstraZeneca, GlaxoSmithKline, and Sanofi; has given lectures/webinars at meetings supported by AstraZeneca, Chiesi, and GlaxoSmithKline; has taken part in clinical trials sponsored by AstraZeneca, GlaxoSmithKline, Novartis, Regeneron, and Sanofi, for which his institution received remuneration; and has current research grants funded by GlaxoSmithKline and a quality improvement grant funded by AstraZeneca.

**Dermot Ryan** has (in the last 3 years) lectured on behalf of, received sponsorship from, or acted as a paid advisor to Mylan, AZ, Chiesi, Novartis, GlaxoSmithKline, Boehringer Ingelheim and Regeneron.

**Celine Bergeron** reports advisory board participation of Sanofi-Regeneron, AstraZeneca, Takeda, ValeoPharma, consultant for Areteia, honorarium for presentations for AstraZeneca/Amgen, GlaxoSmithKline, Grifols, Sanofi-Regeneron, ValeoPharma and grants paid to The University of British Columbia from BioHaven, Sanofi-Regeneron, AstraZeneca, and GlaxoSmithKline.

**Mona S. Al-Ahmad** has received advisory board and speaker fees from AstraZeneca, Sanofi, Novartis, and GlaxoSmithKline, and received a grant from Kuwait Foundation for the Advancement of Sciences (KFAS).

**Delbert R. Dorscheid** is on faculty at the University of British Columbia and is supported by the following grants: Canadian Institutes of Health Research, British Columbia Lung Association, and Michael Smith Foundation for Health Research. In addition, he has received speaking fees, travel grants, unrestricted project grants, writing fees and is a paid consultant for Pharma including Sanofi Regeneron, Novartis Canada, AstraZeneca, GlaxoSmithKline and ValeoPharma. He is an active member of Canadian Thoracic Society, American Thoracic Society, European Respiratory Society, and the Allergen Research Network. Dr. Dorscheid does not believe that any of the disclosed potential conflicts represent true conflicts with respect to the information and recommendations included in this manuscript.

**Eileen Wang** has received honoraria from AstraZeneca, GlaxoSmithKline, Amgen, and Genentech. She has been an investigator on studies sponsored by AstraZeneca, GlaxoSmithKline, Genentech, and Sanofi, for which her institution has received funding.

**John D. Blakey** has received grants or contracts from Asthma Australia, MRFF, FHRI, Telethon Kids Institute, International Primary Care Respiratory Group, and the Charlies Foundation for Research. He has received payment or honoraria for lectures, presentations, or speaker bureaus from Chiesi, The Limbic, Boehringer Ingelheim, GlaxoSmithKline, AstraZeneca, and Sanofi. He has received support for attending meetings and/or travel from GlaxoSmithKline, Centre for Research Excellence in Treatable Traits, AstraZeneca, and The George Institute. He has participated in the study steering committee of GlaxoSmithKline, unrelated to this work. He has taken a leadership role in the Thoracic Society of Australia and New Zealand, and Asthma Australia. He has received medical writing/equipment from GlaxoSmithKline, and Novartis.

**Belinda Cochrane** has received advisory fees from GlaxoSmithKline and Sanofi, speaker fees from AstraZeneca, Moderna and Chiesi, and research grant funding from GlaxoSmithKline.

**Matthew J. Peters** declares personal fees and non-financial support from AstraZeneca, GlaxoSmithKline, and Sanofi.

**Todor A. Popov** declares research support from Novartis and Chiesi Pharma, not related to this particular work.

**Carlos A. Torres-Duque** has received fees as advisory board participant and/or speaker from AstraZeneca, Boehringer-Ingelheim, GlaxoSmithKline, Novartis, and Sanofi-Aventis; has taken part in clinical trials from AstraZeneca, Novartis and Sanofi-Aventis; has received unrestricted grants for investigator-initiated studies at Fundacion Neumologica Colombiana from AstraZeneca, Boehringer-Ingelheim, GlaxoSmithKline, Grifols, and Novartis.

**Susanne Hansen** declares no relevant conflict of interest.

**Francesca Puggioni** reports having received lectures or advisory board fees from Menarini, Mundipharma, Chiesi, Alk Abello, AstraZeneca, Boehringer Ingelheim, Guidotti, Malesci, GlaxoSmithKline, Hal Allergy, Novartis, Sanofi, Regeneron, Stallergenes Greer, Valeas, and Almirall.

**Kirsty Fletton** is an employee of Optimum Patient Care Global (OPCG), a co-funder of the International Severe Asthma Registry.

**Laila Salameh** declares no relevant conflict of interest.

**Peter G. Middleton** declares grant support from HCF Australia, Canadian Institutes of Health, Board membership of the CF Foundation DSMB, and lecture fees and advisory boards for AstraZeneca, Limbic, MedEd, and Vertex, all unrelated to this work.

**Paulo Márcio Pitrez** received fees as a speaker or for consultations from GlaxoSmithKline, AstraZeneca, Sanofi, and Aché.

**Chin Kook Rhee** received consulting/lecture fees from Merck Sharp & Dohme, AstraZeneca, GlaxoSmithKline, Novartis, Takeda, Mundipharma, Boehringer-Ingelheim, Teva, Sanofi, Organon, Roche, and Bayer.

**Eve Denton** declares grants to her institution from AstraZeneca, GlaxoSmithKline, Novartis, Sanofi, Teva, and Seqirus, for unrelated projects and speaker fees from Sanofi, and Boehringer Ingelheim.

**Kenneth R. Chapman** has received grants from AstraZeneca, Boehringer Ingelheim, Bellus, CSL Behring, GlaxoSmithKline, Grifols, Inhibrx, Novartis, Regeneron, Sanofi, Takeda, Vertex, consulting fees from AstraZeneca, CSL Behring, GlaxoSmithKline, Grifols, Inhibrx, Novartis, Sanofi, Takeda. He has a leadership or fiduciary role in Alpha-1 Canada, Canadian Thoracic Society, Alpha-1 Foundation, and AlphaNet Canada.

**Lauri Lehtimäki** has received personal fees from ALK, AstraZeneca, Berlin Chemie, Boehringer Ingelheim, Chiesi, GlaxoSmithKline, Menarini, Novartis, Orion Pharma and Sanofi.

**Ruth B. Murray** is a consultant for Observational and Pragmatic Research Institute (OPRI) which conducted this study in collaboration with Optimum Patient Care, a co-funder of the International Severe Asthma Registry.

**Chau-Chyun Sheu** has received advisory board and speaker fees from AstraZeneca, Boehringer Ingelheim, GlaxoSmithKline, Novartis, and Pfizer, and has acted as an investigator for trials sponsored by AstraZeneca, Novartis, Roche, Sanofi-Regeneron, Galapagos, Shionogi, Aridis, Bristol Myers Squibb, Insmed, United Therapeutics, Enanta Pharmaceuticals, Areteia Therapeutics, Meiji, and Horizon Therapeutics.

**David J. Jackson** has received speaker fees and consultancy fees from AstraZeneca, GlaxoSmithKline, Sanofi Regeneron, and Boehringer Ingelheim, and research funding from AstraZeneca.

**Riyad Al-Lehebi** has given lectures at meetings supported by AstraZeneca, Boehringer Ingelheim, Novartis, GlaxoSmithKline, and Sanofi, and participated in advisory board fees from GlaxoSmithKline, AstraZeneca, Novartis, and Abbott.

**Mariko Siyue Koh** reports grant support from AstraZeneca, and honoraria for lectures and advisory board meetings paid to her hospital (Singapore General Hospital) from GlaxoSmithKline, AstraZeneca, Novartis, Sanofi, and Boehringer Ingelheim, outside the submitted work.

**Bassam Mahboub** reports no conflict of interest.

**Ledit R. F. Adrusso** is an investigator and/or speaker for AstraZeneca, GlaxoSmithKline, Novartis, Sanofi, Amgen, Chiesi, Bellus, Areteria, Insmed, and Optinose.

**Athena Gogali** received honoraria for presentations and consultancy fees from: AstraZeneca, Boehringer Ingelheim, Chiesi, ELPEN, GlaxoSmithKline, Novartis, and Menarini.

**Giorgio Walter Canonica** has received research grants, as well as lecture or advisory board fees from A. Menarini, Alk-Albello, Allergy Therapeutics, Anallergo, AstraZeneca, MedImmune, Boehringer Ingelheim, Chiesi Farmaceutici, Circassia, Danone, Faes, Genentech, Guidotti Malesci, GlaxoSmithKline, Hal Allergy, Merck, Merck Sharp & Dohme, Mundipharma, Novartis, Orion, Sanofi Aventis, Sanofi, Genzyme/Regeneron, Stallergenes, UCB Pharma, Uriach Pharma, Teva, Thermo Fisher, and Valeas.

**Piotr Kuna** reports personal fees from Adamed, AstraZeneca, Berlin Chemie Menarini, FAES, Glenmark, Novartis, Polpharma, Boehringer Ingelheim, Teva, Zentiva, outside the submitted work.

**Martin Sivori** has received lecture fees for medical education programs of AstraZeneca, GlaxoSmithKline, and TEVA.

**Renaud Louis** has received grants and lecture fees from AstraZeneca, GlaxoSmithKline, and Sanofi.

**Shelley Abercromby** declares no relevant conflict of interest.

**Giuseppe Guida** received lecture fees from GlaxoSmithKline and AstraZeneca.

**Bernt Bøgvald Aarli** reports grants from AstraZeneca and Novartis, honoraria for presentations from AstraZeneca, GlaxoSmithKline, Sanofi-Aventis, and Orion, consulting fees from AstraZeneca, Grifols, and IM Medical Education Nordic AB, and has participated in advisory boards for AstraZeneca, GlaxoSmithKline, Chiesi, Sanofi-Aventis, Grifols, Orion, and Celltrion Healthcare, and he reports owning stocks in KBB Medic (Medical app company), outside the submitted work. His institution has received funding from Novartis and AstraZeneca.

**Aaron Beastall** is an employee of Optimum Patient Care Global, a co-funder of the International Severe Asthma Registry.

**Victoria Carter** is an employee of Optimum Patient Care (OPC). OPC is a co-funder of the International Severe Asthma Registry.

**Ghislaine Scelo** is a consultant for Observational and Pragmatic Research Institute (OPRI). OPRI conducted this study in collaboration with Optimum Patient Care, a co-funder of the International Severe Asthma Registry.

**John Townend** is an employee of the Observational and Pragmatic Research Institute (OPRI). OPRI conducted this study in collaboration with Optimum Patient Care, a co-funder of the International Severe Asthma Registry.

**Borja G. Cosio** declares grants from Chiesi, Menarini, and GlaxoSmithKline; personal fees for advisory board activities from Chiesi, GlaxoSmithKline, Novartis, Sanofi, Teva, and AstraZeneca; and payment for lectures/speaking engagements from Chiesi, Novartis, GlaxoSmithKline, Menarini, and AstraZeneca, outside the submitted work.

**Pujan H. Patel** has received advisory board and speaker fees from AstraZeneca, GlaxoSmithKline, Novartis, and Sanofi/Regeneron.

**Celine Yun Yi Goh** is an employee of Optimum Patient Care Global (OPCG), a co-funder of the International Severe Asthma Registry.

**Zsuzsanna Csoma** declares no relevant conflict of interest.

**John W. Upham** has received speaker and consulting fees from Novartis, AstraZeneca, GlaxoSmithKline, Sanofi, and Boehringer Ingelheim.

**João A. Fonseca** reports grants from research agreements with AstraZeneca, Mundipharma, Sanofi Regeneron, and Novartis. Personal fees for lectures and attending advisory boards: AstraZeneca, GlaxoSmithKline, Mundipharma, Novartis, Sanofi Regeneron, and Teva.

**Peter G. Gibson** has received speaker fees and grants to his institution from AstraZeneca, GlaxoSmithKline, and Novartis.

**Christine Jenkins** is a member of advisory boards for AstraZeneca, GlaxoSmithKline and Chiesi. She has received payment for Advisory board attendance, honoraria, lectures and meeting participation as a speaker. She has no conflicts in relation to this work.

**Guy G. Brusselle** has received honoraria for lectures from AstraZeneca, Boehringer Ingelheim, Chiesi, GlaxoSmithKline, and Novartis. He is a member of advisory boards for AstraZeneca, Boehringer Ingelheim, GlaxoSmithKline, Merck Sharp & Dohme (MSD), Novartis, and Sanofi/Regeneron.

**Anne Chèvremont** declares no conflict of interest.

**Andréanne Côté** declares she has received speaking fees and consultant fees from Sanofi, Regeneron, AstraZeneca, and GlaxoSmithKline. She received unrestricted grant support from GlaxoSmithKline, and AstraZeneca.

**Carlos Andrés Celis-Preciado** declares no relevant conflict of interest.

**Ivan Solarte** has received fees as an advisory board participant and/or speaker from GlaxoSmithKline, AstraZenca, and Sanofi.

**Celeste M. Porsbjerg** has attended advisory boards for AstraZeneca, Novartis, TEVA, and Sanofi-Genzyme; has given lectures at meetings supported by AstraZeneca, Novartis, TEVA, Sanofi-Genzyme, and GlaxoSmithKline; has taken part in clinical trials sponsored by AstraZeneca, Novartis, Merck Sharp & Dohme, Sanofi-Genzyme, GlaxoSmithKline, and Novartis; and has received educational and research grants from AstraZeneca, Novartis, TEVA, GlaxoSmithKline, ALK, and Sanofi-Genzyme.

**Asger Sverrild** has attended advisory boards for Sanofi, and GSK, has given lectures at meetings supported by AstraZeneca, GSK, and Sanofi, and has taken part in clinical trials sponsored by AstraZeneca, Sanofi, and Eli Lilly.

**Paula Kauppi** has received lecture fees from GlaxoSmithKline, consultancy fees from Sobi, and PI fees from Theravance.

**Stelios Loukides** has received fees and honoraria from Menarini, GlaxoSmithKline, Novartis, Elpen, Pfizer, Gilead, Guidotti, AstraZeneca, and Chiesi.

**Michael P. Makris** reports honoraria for presentations and consultancy fees from Novartis, GlaxoSmithKline, Menarini, AstraZeneca, Chiesi, Sanofi, Pfizer, outside the submitted work.

**Andriana I. Papaioannou** has received fees and honoraria from Menarini, GlaxoSmithKline, Novartis, Elpen, Boehringer Ingelheim, AstraZeneca, Demo, and Chiesi.

**Enrico Heffler** declares personal fees for advisory boards participation and/or speaker activities from: Sanofi, Regeneron, GlaxoSmithKline, Novartis, AstraZeneca, Stallergenes-Greer, Circassia, Bosch, Celltrion-Healthcare, Chiesi, and Almirall.

**Jeffrey Shi Kai Chan** is an employee of the Observational and Pragmatic Research Institute (OPRI). OPRI conducted this study in collaboration with Optimum Patient Care, a co-funder of the International Severe Asthma Registry.

**Hyonsoo Joo** reports no conflict of interest.

**Liam G. Heaney** has received grant funding, participated in advisory boards and given lectures at meetings supported by Amgen, AstraZeneca, Boehringer Ingelheim, Chiesi, Circassia, Hoffmann la Roche, GlaxoSmithKline, Novartis, Theravance, Evelo Biosciences, Sanofi, and Teva; he has received grants from MedImmune, Novartis UK, Roche/Genentech Inc, GlaxoSmithKline, Amgen, Genentech/Hoffman la Roche, AstraZeneca, MedImmune, Aerocrine, and Vitalograph; he has received sponsorship for attending international scientific meetings from AstraZeneca, Boehringer Ingelheim, Chiesi, GlaxoSmithKline, and Napp Pharmaceuticals; he has also taken part in asthma clinical trials sponsored by AstraZeneca, Boehringer Ingelheim, Hoffmann la Roche, and GlaxoSmithKline for which his institution received remuneration; he is the Academic Lead for the Medical Research Council Stratified Medicine UK Consortium in Severe Asthma which involves industrial partnerships with a number of pharmaceutical companies including Amgen, AstraZeneca, Boehringer Ingelheim, GlaxoSmithKline, Hoffmann la Roche, and Janssen.

**Wei-Han Cheng** was an employee of Sanofi and may hold stock and/or stock options in the company.

**Njira Lugogo** received consulting fees from Amgen, AstraZeneca, Avillion, Genentech, GSK, Niox, Novartis, Regeneron, Sanofi, and Teva; honoraria for non-speakers bureau presentations from GSK, TEVA and Astra Zeneca; and travel support from Astra Zeneca, SANOFI, TEVA, Regeneron and GSK; her institution received research support from Amgen, AstraZeneca, Avillion, Bellus, Evidera, Gossamer Bio, Genentech, GSK, Janssen, Niox, Regeneron, Sanofi, Novartis and Teva. She is an honorary faculty member of Observational and Pragmatic Research Institute (OPRI) but does not receive compensation for this role.

**Michael E. Wechsler** reports grants and/or personal fees from Novartis, Sanofi, Regeneron, Genentech, Sentien, Restorbio, Equillium, Genzyme, Cohero Health, Teva, Boehringer Ingelheim, AstraZeneca, Amgen, GlaxoSmithKline, Cytoreason, Cerecor, Sound Biologics, Incyte, and Kinaset.

**Cláudia Chaves Loureiro** has received (in the last 3 years) lecture or advisory board fees from AstraZeneca, GlaxoSmithKline, Menarini, Sanofi, and Teva, outside this work.

**Bellanid Rodríguez-Cáceres** declares no relevant conflict of interest.

**Tatsuya Nagano** received lecture fees from Kyorin, Sanofi, GlaxoSmithKline, Novartis, and AstraZeneca.

**Zhixiao Wang** is an employee of Regeneron Pharmaceuticals, Inc, and holds stock and/or stock options in the company.

**Hao-Chien Wang** reports no conflict of interest.

**Jorge Máspero** reports speaker fees, grants, or advisory boards for AstraZeneca, Sanofi, GlaxoSmithKline, Novartis, Inmunotek, Menarini, and Noucor.

**Fernando Saldarini** is a speaker of GlaxoSmithKline, AstraZeneca, and SANOFI.

**Ana María Stok** has acted as an investigator for GlaxoSmithKline, AstraZeneca, Sanofi, Chiesi, Novartis, and Bago. She reports speaker fees from GlaxoSmithKline, AstraZeneca, and Sanofi.

**Anahi Yañez** has received grants/research supports from GlaxoSmithKline, AstraZeneca, Sanofi, Chiessi, Novartis, MDS, Roche, Faes, TEVA, Avillon, Janssen, Bayer, Sanofi Gynzene, and he has received consultation fees from GlaxoSmithKline, AstraZeneca, Sanofi, and Eurofarma. He has participated in a company sponsored speaker's bureau from Sanofi, GlaxoSmithKline, Faes, Sanofi Gynzene, and AstraZeneca.

**Philip G. Bardin** declares no relevant conflict of interest.

**Sinthia Z. Bosnic-Anticevich** has received honorarium for participation in expert advisory boards and given lectures for Teva Pharmaceuticals, AstraZeneca, GlaxoSmithKline, Meda/Mylan, Sanofi, Mylan, Chiesi, Menarini, Sanofi, Boehringer Ingelheim, Abbvie and received unrestricted research grants from Mylan, AstraZeneca, Teva, AstraZeneca, GlaxoSmithKline, and Viatris.

**Vidya Navaratnam** was an employee of OPCA when she worked on this manuscript, but she has left OPCA. She has received payment for lectures/speaking engagements from Boehringer Ingelheim as well as payment for travel/accommodation/meeting expenses from Boehringer Ingelheim and Bristol Myer Squibs.

**Mohit Bhutani** has received speaker and consultant fees for AstraZeneca, GlaxoSmithKline, Sanofi, Covis, Boerhinger Ingelheim, Takeda and Valeo. He has leadership roles within the Canadian Thoracic Society.

**M. Diane Lougheed** has (in the past 3 years) received grants outside the submitted work paid directly to Queen's University from the Canadian Institutes of Health Research (sub-grant from Ottawa Health Research Institute), Manitoba Workers Compensation Board, Ontario Lung Association, Ontario Thoracic Society, the Government of Ontario's Innovation Fund, Queen's University, Astra Zeneca and GlaxoSmithKline; and honoraria from the Canadian Thoracic Society for co-development and co-presentation of as Severe Asthma PREP course, from MD Breifcase for co-development of an accredited CME module on Severe Asthma; and from AstraZeneca for participation in the Precision Program Advisory Board. She has also served as a member and past-chair of the Canadian Thoracic Society (CTS) Asthma Clinical Assembly, member of the CTS Asthma Clinical Assembly Steering Committee, CTS representative on the Lung Association's Board of Directors, CTS representative to the European Respiratory Society, and member of Health Quality Ontario's Asthma in Adults and Asthma in Children Quality Standard Advisory Committee.

**Lyle Melenka †** declares no conflict of interest.

**Petros Bakakos** declares no conflict of interest.

**Konstantinos P. Exarchos** declares no relevant conflict of interest.

**Aggelos A. Ladias** declares no conflict of interest.

**Dóra Lúdvíksdóttir** has received lecture fees from GlaxoSmithKline, Sanofi and AstraZeneca.

**Takashi Iwanaga** received speaker bureau fees from Kyorin, GlaxoSmithKline, Novartis, Boehringer Ingelheim, AstraZeneca, and Sanofi.

**Elvia Angelica Contreras Contreras** declares no relevant conflict of Interest.

**Sverre Lehmann** has been an investigator on clinical trials sponsored by GlaxoSmithKline and AstraZeneca, for which his institution has received funding.

**José Alberto Ferreira** declares no conflict of interest.

**Rebecca Gall** is an employee and shareholder of Regeneron Pharmaceuticals, Inc.

**Pin-Kuei Fu** declares no relevant conflict of interest.

**Diahn-Warng Perng** received sponsorship to attend or speak at international meetings, honoraria for lecturing or attending advisory boards, and research grants from the following companies: AstraZeneca, Boehringer Ingelheim, GlaxoSmithKline, Novartis, Daiichi Sankyo, Shionogi, and Orient Pharma.

**Flavia Hoyte** declares honoraria from AstraZeneca and Genentech. She has been an investigator on clinical trials sponsored by GlaxoSmithKline, Genentech, Teva, Sanofi, for which her institution has received funding.

**Rohit Katial** declares no relevant conflict of interest.

**Unnur S. Björnsdóttir** receives gratuities for lectures/presentations from AstraZeneca, Sanofi and Novartis.

**Camille Taillé** has received lecture or advisory board fees and grants to her institution from AstraZeneca, Sanofi, GlaxoSmithKline, Chiesi, Stallergenes, Celltrion and Novartis, for unrelated projects.

**Christian Taube** declares no relevant conflict of interest.

**Breda Cushen** has received honoraria for lectures and received sponsorship for attending meetings from AstraZeneca, Novartis, and Boehringer Ingelheim. She has participated in advisory boards, and provided consultancy,for Chiesi and Sanofi.

**Lakmini Bulathsinhala** is an employee of the Observational and Pragmatic Research Institute (OPRI). OPRI conducted this study in collaboration with Optimum Patient Care, a co-funder of the International Severe Asthma Registry.

**Leif Bjermer** has (in the last 3 years) received lecture or advisory board fees from Alk-Abello, AstraZeneca, Chiesi, GlaxoSmithKline, Sanofi, and Genzyme/Regeneron.

**David B. Price** has advisory board membership with AstraZeneca, Boehringer Ingelheim, Chiesi, GlaxoSmithKline, Novartis, Viatris, Teva Pharmaceuticals; consultancy agreements with AstraZeneca, Boehringer Ingelheim, Chiesi, GlaxoSmithKline, Novartis, Viatris, Teva Pharmaceuticals; grants and unrestricted funding for investigator-initiated studies (conducted through Observational and Pragmatic Research Institute Pte Ltd) from AstraZeneca, Chiesi, Viatris, Novartis, Regeneron Pharmaceuticals, Sanofi Genzyme, and UK National Health Service; payment for lectures/speaking engagements from AstraZeneca, Boehringer Ingelheim, Chiesi, Cipla, Inside Practice, GlaxoSmithKline, Medscape, Viatris, Novartis, Regeneron Pharmaceuticals and Sanofi Genzyme, Teva Pharmaceuticals; payment for travel/accommodation/meeting expenses from AstraZeneca, Boehringer Ingelheim, Novartis, Medscape, Teva Pharmaceuticals.; owns 74% of the social enterprise Optimum Patient Care Ltd (Australia and UK) and 92.61% of Observational and Pragmatic Research Institute Pte Ltd (Singapore); is peer reviewer for grant committees of the UK Efficacy and Mechanism Evaluation Programme, and Health Technology Assessment; and he was an expert witness for GlaxoSmithKline.
