# Supplementary material for: Biomarker profile and disease burden associated with intermittent and long-term oral corticosteroid use in patients with severe asthma prior to biologic initiation in real-life (STAR)
Source: World Allergy Organ J. 2025 Jun 3;18(7):101066. doi: 10.1016/j.waojou.2025.101066 (PMC12169735; doi:10.1016/j.waojou.2025.101066)
Supplement: Multimedia component 1 [file mmc1.docx]

# Online Supplement

**Online methods**

**International Severe Asthma Registry**

- All data collection sites in ISAR have obtained regulatory agreement in compliance with specific data transfer laws, country-specific legislation, and relevant ethical boards and organizations.^1^
- The ISAR database has ethical approval from the Anonymous Data Ethics Protocols and Transparency (ADEPT) committee (ADEPT0218) and is registered with the European Union Electronic Register of Post-Authorization studies (ENCEPP/DSPP/23720).
- The study was designed, implemented, and reported in compliance with the European Network Centres for Pharmacoepidemiology and Pharmacovigilance (ENCEPP) Code of Conduct (EMA 2014; EUPAS49201) and with all applicable local and international laws and regulation, and registered with ENCEPP (https://www.encepp.eu/encepp/viewResource.htm?id=48848). Governance was provided by ADEPT (registration number: ADEPT1022).

**Working Group Members**

**Argentina:** María Eugenia Franchi, Ana Giselle Tomaszuk.

**India:** Sundeep Salvi.

**Ireland:** Richard W. Costello.

**Portugal**: Ana Sá-Sousa.

**International Severe Asthma Registry (ISAR) Collaborators**

**Argentina:** Vanessa Abrate, Matías Ardusso, Gabriela Chirino, Mónica De Gennaro, Romina Fernandez, Yasmin García-Castañeda, Marcos Hernandez, Veronica Lawriwskyj, Diego Litewka, Maria Orazi, Ileana Palma, Josefina Pascua, Carla Ritchie, Ramón Rojas, Fernando Ariel Serrano, Evelyn Sureda, Alejandro Videla.

**AstraZeneca:** Lyra Agustin, Chris Brooks, Eileen Dareng, Alexander de Giorgio-Miller, Benjamin Emmanuel, Cathy Emmas, Hisham Farouk, Robert Fogel, Sachin Ravinda Joshi, Rafal Kucharski, Justin Kwiatek, Carrie Lancos, Tham T. Le, Andrew N. Menzies-Gow, David Peters, Dawn Ruff, Neda Stjepanovic, Trung N. Tran, Hannah Urbanski, Lee Wulund.

**Australia:** Li Ping Chung, Graham Hall, Rebecca Hetherington, Gregory P. Katsoulotos, David Langton, Joanna Lee, Bharvi Maneck, Laura Mitchell, Natasa Petrovic, Paul Reynolds, Hayley See, Vincent So, Rachel Tan.

**Belgium:** Virginie Paulus.

**Brazil:** Adyléia Aparecida Dalbo Contrera Toro, Marcos Antunes, Daniela Blanco, Lilian Caetano, Débora Carla Chong-Silva, Amanda da Rocha Olveira Cardoso, Maria Enedina de Aquino Scuarcialupi, Luciana de Freitas Veloso Monte, Kamila Ticiana Dias Ferreira, José Elabras Filho, Milena Baptistella Grotta, Marina Lima, Paulo José Cauduro Marostica, Desiree Jacob Monteiro, Maria Ines Perello, Marcia Pizzichini, Luiz Vicente Ribeiro Ferreira da Silva Filho, José Gustavo Barian Romaldini, Adalberto Sperb Rubin, Lúcia Helena Messias Sales, Carolina Barbosa Souza Santos, Faradiba Sarquis Serpa, Matheus Augusto Nunes Ventura.

**Bulgaria:** Plamen Hristov Yakovliev, Diana X. Hristova, Cvetanka Hristova Odzhakova, Miroslav Ivanov Stamenov, Mariana Mandajieva, Sonya Metodieve Genova, Violina Milchova Vasileva, Darina Petrova Dimova, Eleonora M. Stamenova, Nadezhda K. Takovska, Michail Todorov, Katya Vasileva Noeva.

**Canada:** Shawn D. Aaron, Hannah Anstruther, Marie-Eve Boulay, Emma Bullock, Kayla Cardoso, Beth Davis, Jane Duke, Martine Duval, Cathy Fugere, Kylie Haydey, Leiana Hoshyari, Angie Johnson, Amy May, Carrie McPhee, Alison Morra, Maria Naval, Ron Olivenstein, Shoshana Parker, Leeanne Parris, Brianne Philipenko, Heather Ryan, Hana Serajeddini, Lindsay Simmonds, Kathy Vandemheen.

**Colombia:** Abraham Alí-Munive, Fabio Bolivar, Christian Chapman, Maria Jose Fernandez Sanchez, Elizabeth García Gomez, Julian Esteban Londoño Hernandez, Luisa Manrique, Jaime Ocampo Gomez, Patricia Parada, Audrey Piotrostanalzki Vargas, Isabella Perna Reyes, Diana Jimena Cano Rosales, Lucy Yaquelin Sanchez Duran, Janeth Rocio Higuera Sarmiento, Leslie K. Vargas-Ramirez.

**Denmark:** Maria Bisgaard Borup, Anne-Sofie Bjerrum, Lycely Dongo, Kjell Erik Julius Håkansson, Ole Hilberg, Sofie Johansson, Claus Rikard Johnsen, Linda M. Rasmussen, Johannes Schmid, Marianne Søndergaard, Niels Steen Krogh, Truls Sylvan Ingebrigtsen, Roxana Vijdea, Anna von Bülow.

**Ecuador:** Juan Carlos Calderon, Ivan Cherrez-Ojeda, Karla Robles, Eunice Robles.

**Estonia:** Marily Jaagor, Kai Kliiman, Pilleriin Liiva, Jana Marinina, Renata Melnikova, Triin Sadam, Svetlana Sergejeva, Liina Viks.

**France:** Karima Bourayou, Abla Chaachoua, Jérémy Charriot, Cecile Chenivesse, Gilles Devoussoux, Candice Estellat, Gilles Garcia, Amal Gouider, Amina Kertobi, Nicolas Roche, Linda Thieulon, Yannick Vacher, Eric Van Ganse.

**Germany:** Ina Haasler, Stephanie Korn.

**Greece:** Xenophon Aggelides, Mina Gaga, Nick Gavogiannakis, Maria Kallieri, Lampros Kalogiros, Dimitris Mitsias, Maria Ntakoula, Anastasia Papaporfuriou, Giannis Paraskevopoulos, Fotis E. Psarros, Agni Sioutkou, Konstantinos Tatsis, Lefteris Zervas.

**India:** Priyanka Dhumal, Swapnil Gadhave, Jyoti Narwadkar.

**Ireland - Beaumont Hospital:** Deirdre Long, Elaine MacHale, Dorothy Ryan.

**Ireland - Tallaght:** Patrick D. Mitchell, Caoimhe Murphy.

**Italy:** Nicola Barbarini, Cristina Cardini, Matteo Gabetta, Concetta Sirena, Morena Stuppia.

**Japan:** Kazuhisa Asai, Chei Choy-Lye, Hajime Fujimoto, Takao Fujisawa, Yuuji Fujita, Hironobu Fukuda, Kana Hamada, Yoshinori Haruta, Masahiro Hirose, Takahiko Horiguchi, Soichiro Hozawa, Yoshikazu Iwasaki, Yoko Kajino, Tetsu Kobayashi, Hisako Matsumo, Mayumi Matsunaga, Yumi Matsuoka, Kimiko Mori, Akio Niimi, Yoshihiro Nishimura, Kazutaka Nogami, Tsuyoshi Oguma, Hiroshi Ohnishi, Kumiko Ota, Kiyoshi Sekiya, Hironobu Sunadome, Tomoko Tajiri, Hiroshi Tanaka, Yuji Tohda.

**Kuwait:** Asmaa Ali, Amr Attiya, Ahmed Maher, Sumi Rajeevan, Wafa Talaat.

**Mexico:** Shagra G. Arana-Berrera, Hugo Alberto Azuara Trujillo, Lilia Margarita Borboa, Ricardo Campos Cerda, Rosa Isela Campos Gutiérrez, Begonia Casas, Nidia Karen Castillon Benavides, Saraid Cerda Reyes, Aurora Alejandra Chavez Garcia, Ana Karina Z. Clavellina, Alberto Correa, Maria de la Luz García, Blanca del Río Navarr, Liliana Dominguez Vaca, Veronica Domínguez Vaca, Miryam Lizet Flores Cruz, Ulises García, Rodrigo Hiroshi Gonzalez Luna, Yair Humberto Gonzalez Tuyub, Victor Gonzalezu, Diana Herrera, Nadia Margarita Hinojosa, Claudia Elizabeth Jiménez Carrillo, Alejandro Jiménez Chobillón, Ana Paola Macias Robles, Laura Dafne Mendoza Reyna, Claudine Isela Nava Ramírez, Elsy Maureen Navarrete, Patricia María O´Farril Romanillos, Itzel Vianney Ochoa García, Karen Lillian Rivera Alvarado, Fernanda Rodríguez Monroy, Francisco Salcedo Rodríguez, Victor Sandoval, Mariano Temix.

**Netherlands:** Elisabeth Bel, Anke-Hilse Maitland-van der Zee, Job F.M. van Boven, Katia M.C. Verhamme.

**Norway:** Sverre Lehmann, Bernt Bøgvald Aarli, Anders Floymo, Ingrid Malling Fløystad, Mads Frigstad, Aida Kvitting, Siri Narum.

**Poland:** Agnieszka Lawkiedraj.

**Portugal:** Paula Maria Alendouro Ribeiro, Ana Maria Arrobas, Ines Belchior, Margarida Borges, Filipa Carriço, Carla Chaves Loureiro, Marta Drummond, Emilia Faria, Ricardo Lima, Carlos Lopes, Cristina Lopes, Ana Mendes, Ana Margarida Pereira, Luís Pereira Amaral, Vânia Catarina Pereira Caldeira, Claudia Sofia Pinto, Paula Leiria Pinto, Frederico Regateiro, Cecilia Rodrigues Pardal, Hadassa Santos, Natacha Santos, Anna Sokolova, Cláudia Sousa, Wanda Videira.

**Respiratory Effectiveness Group:** Mina Gaga, Graham Lough, Valeria Perugini, Michael Walker.

**Saudi Arabia:** Walaa Abuzahra, Salama Ahmed, Abdalla Alasiri, Hamdan AlJahdali, Lujain Alshaigi, Julmilyn Arnuco, Ma Carla Gimoro, Yahya Habis, Amr Salah, Siraj Wali.

**Singapore:** John Arputhan Abisheganaden, Eileen Chew, Sanjay Chotirmall, Tavleen Kaur Jaggi, Mei Fong Liew, Pee Hwee (Esther) Pang, Tze Lee Tan, Tunn Ren Tay, Augustine Tee.

**South Korea:** Jae Ha Lee, Seung Won Ra, Kwang Ha Yoo.

**Spain:** Jose Antonio Gullón, Eva Martinez-Moragón, Isabel Urrutia, Cristina Vega.

**Taiwan:** Ling-Yi Chang, Joanna Chen, Kuan-Yuan Chen, Xiang Ying Chen, Shih-Lung Cheng, Ying-Chun Chien, Kuo-Chin Chiu, Chu-Kuang Chou, Yi Rou Du, Yun Rui Fu, Liang-Wen Hang, Yuan Zhen Hong, Meng-Jer Hsieh, Jeng-Yuan Hsu, Erick Wan-Chun Huang, Hsin-Kuo Ko, Kang-Yun Lee, Shu Wen Lee, Xiao Ting Lee, Rong Ru Lim, Ching-Hsiung Lin, Horng-Chyuan Lin, Ming-Shian Lin, Sheng-Hao Lin, Shih-Feng Liu, Jia Yi Ng, Pei Jun Ou, Sheng-Yeh Shen, Yi Jun Shi, Wan Ru Wong, Cheng Hui Xu, Yu Qiao Zheng.

**United Kingdom:** John Busby.

**USA - National Jewish Health:** Jennifer Brandorff, Nicholas Chapman, Jessica Cummings, Amanda Grippen Goddard, Christena Kolakowski, Jacqui Marti, Kanao Otsu, Robert Schell.

**USA - University of Michigan:** Amirbehzad Bagheri, Raul Desiderio, Michael Hadden, Hannah Harwood, Pam James, Arjun Mohan.

**USA - University of North Carolina:** Stephen Schworer, Stephen Tilley.

**USA - University of Texas Health San Antonio:** Diego J. Maselli.

**Supplemental Tables**

**S-Table 1:** Time from latest biomarker measurements (most recent at or before biologic initiation) to biologic initiation.

**Interval (days) from latest BEC measurement to biologic initiation**

Group | N Median (IQR) Min Max

-----------------+------------------------------------------------------------

No OCS | 150 24 0 133 0 364

Intermittent OCS | 1460 19 0 99 0 365

Long-term OCS | 1288 0 0 63 0 365

------------------------------------------------------------------------------

**Interval (days) from latest FeNO measurement to biologic initiation**

Group | N Median (IQR) Min Max

-----------------+------------------------------------------------------------

No OCS | 126 5 0 56 0 355

Intermittent OCS | 1044 0 0 29 0 364

Long-term OCS | 939 0 0 8 0 364

------------------------------------------------------------------------------

**Interval (days) from latest IgE measurement to biologic initiation**

Group | N Median (IQR) Min Max

-----------------+------------------------------------------------------------

No OCS | 145 5 0 72 0 365

Intermittent OCS | 1272 13 0 95 0 365

Long-term OCS | 778 18 0 108 0 359

------------------------------------------------------------------------------

N = number of patients with data within each group. Median (inter-quartile range), minimum and maximum values are in days between the most recent biomarker measurement and biologic initiation.

**S-Table 2: Study variables**

| **Patient demographics** | |
| --- | --- |
| GINA 2018 Treatment Step | GINA 2018 Treatment Step at ISAR enrolment: Step 4 with uncontrolled asthma, or Step 5 |
| Age | Age in completed years at the index date |
| Sex | Female or Male |
| BMI | The ratio of weight (kg) to squared height (m^2^). Categorized as underweight (<18.5 kg/m^2^), normal weight (≥18.5 kg/m^2^ and <25 kg/m^2^), overweight (≥25 kg/m^2^ and <30 kg/m^2^), and obese (≥30 kg/m^2^) |
| Age of Asthma Onset | Age in completed years or months (if less than 1 year) at which asthma symptoms began. Early onset: <18 years of age; adult onset: ≥18 years of age |
| Smoking Status | Categorized as non-smoker, current smoker, or ex-smoker (most recent status before starting biologics) |
| **Biomarkers (most recent available measurements before and on the date of biologic initiation)** | |
| BEC | Count of blood eosinophils (cells/µL). Categorized as: BEC <150 cells/µL, BEC ≥150 - <300 cells/µL, BEC ≥300 - <500 cells/µL, and BEC ≥500 cells/µL *(Objective 1 only)* |
| Blood total IgE level | Blood level IgE (IU/mL). Categorized as: IgE <30 IU/mL, IgE ≥30 - <75 IU/mL, and IgE ≥75 IU/mL |
| FeNO | Measurements of FeNO, measured in parts per billion (ppb) at a flow rate of 50 mL/s. Categorized as: FeNO <20 ppb and FeNO ≥20 ppb; FeNO <25 ppb, and FeNO ≥25 ppb |
| **Lung function and allergen tests (most recent lung function and allergen test information before and on the date of biologic initiation)** | |
| ppFEV_1_ | Measured FEV_1_ as % of Predicted value of FEV the first second of expiration (L) |
| Post-bronchodilator FEV_1_/FVC Ratio | Measured FEV_1_ as a ratio of measured FVC |
| Skin Prick Test | Positive skin prick test Allergens included: grass mix, trees, weed mix, aspergillus, mould mix, food mix, dust mite, animal mix, cat hair, and dog hair |
| Serum Allergen Test | Positive serum allergen test  Allergens included: dust mite, grass mix, cat hair, mould mix, dog hair, and aspergillus |
| **Therapy in addition to ICS/LABA (at the index date [date of biologic initiation])** | |
| Anti-IgE | Prescription for Anti-Immunoglobulin E (Anti-IgE) |
| Anti-IL5 | Prescription for Anti-Interleukin 5 (Anti-IL5/5R) |
| Anti-IL4R | Prescription for Anti-IL4R |
| Anti-TSLP | Prescription for Anti-TSLP |
| LAMA | Prescription for LAMA (add-on therapy to ICS/LABA) |
| LTRA | Prescription for LTRA (add-on therapy to ICS/LABA) |
| LAMA + LTRA | Prescription for LAMA + LTRA (add-on therapy to ICS/LABA) |
| Theophylline | Prescription for theophylline (add-on therapy to ICS/LABA) |
| Macrolides | Prescription for a macrolide antibiotic as an add-on therapy |
| LTOCS | Daily dose of long-term (maintenance) oral corticosteroids (OCS) - OCS use for a duration of >90 days before the index date |
| Cumulative dose of OCS in the last 90 days | Label Dose X Frequency x 90 days of LTOCS *(For LTOCS users only)* and rescue steroids in the 90 days before the index date |
| Total dose of OCS | Label Dose X Frequency X Duration of Use for LTOCS and rescue steroids in the year before the index date |
| **Asthma control** | |
| Number of asthma exacerbations | Number of exacerbations requiring rescue steroids in the year prior to index date |
| Asthma control in the past 4 weeks | Categorized as controlled, partly controlled, or uncontrolled according to the GINA Asthma Control Criteria,^2^ ACQ-6,^3^ or ACT^4^ |
| **Healthcare resource utilization** | |
| Number of hospital admissions for asthma | Number of hospital admissions for asthma in the year prior to index date |
| Number of emergency room visits for asthma | Number of emergency room visits for asthma in the year prior to index date |

*Abbreviations*

ACQ : asthma control questionnaire; ACT : asthma control test ; BEC : blood eosinophil count ; BMI : body mass index ; FeNO : fractional exhaled nitric oxide ; FEV_1_ : forced espiratory volume in one second ; FVC : forced vital capacity ; GINA : Global Initiative for Asthma ; ICS : inhaled corticosteroids ; IgE : immunoglobulin E ; IL : interleukin ; LABA : long-acting β_2_-agonist ; LAMA : long-acting muscarinic antagonist ; LTOCS : long-term oral corticosteroid ; LTRA : leukotriene receptor antagonist ; OCS : oral corticosteroid ; ppFEV_1_ : percent predicted forced expiratory volume in one second ; TSLP : thymic stromal lymphopopoietin

Description of eosinophilc phenotype gradient algorithm

The likelihood of eosinophilic phenotype was categorized using a predefined gradient eosinophilic

algorithm based on highest BEC, long-term oral corticosteroid use, elevated fractional

exhaled nitric oxide, nasal polyps, and adult-onset asthma **(S-Table 3**).^5^

**S-Table 3: eosinophilic phenotype gradient algorithm**

| Likelihood of Eosinophilic phenotype | Descriptors |
| --- | --- |
| Grade 3 : most likely | - BEC ≥ 300 cells/µL, OR - Receiving anti-IL5 treatment, OR - Receiving LTOCS AND BEC ≥150 and <300 cells/µL, OR - NOT receiving LTOCS AND BEC ≥150 <300 cells/µL AND ≥2 out of: presence of NP, FeNO ≥25 ppb, adult-onset disease |
| Grade 2 : Likely | - Receiving LTOCS AND BEC <150 cells/µL, OR - NOT receiving LTOCS AND BEC ≥150 and <300 cells/µL AND 1 of: presence of NP, FeNO ≥25 ppb, adult-onset disease |
| Grade 1 : least likely | - NOT receiving LTOCS AND BEC ≥150 and <300 cells/µL AND Without NP, FeNO ≥25 ppb or adult-onset disease, OR - NOT receiving LTOCS AND BEC <150 cells/µL AND 1 of : presence of NP, FeNO ≥25 ppb or adult onset disease |
| Grade 0 : unlikely/noneosinophilic | - NOT receving LTOCS AND BEC <150 cells/µL Without presence of NP, FeNO ≥25 ppb or adult onset disease |

BEC: blood eosinophil count; FeNO: fractionale exhaled nitric oxide; LTOCS: long-term oral corticosteroid; NP: nasal polyps

**S-Table 4: Proportion of patients included in the study who initiated biologics by year and biologic class**

|  | **Biologic class** | | | | | | | |
| --- | --- | --- | --- | --- | --- | --- | --- | --- |
|  | **Anti-IgE** | | **Anti-IL4** | | **Anti-IL5/5R** | | **Anti-TSLP** | |
| **Year** | **N** | **%** | **N** | **%** | **N** | **%** | **N** | **%** |
| pre-2017 | 527 | 71.4 | 0 | 0.0 | 211 | 28.6 | 0 | 0.0 |
| 2017 | 203 | 27.8 | 6 | 0.8 | 522 | 71.4 | 0 | 0.0 |
| 2018 | 163 | 22.1 | 7 | 0.9 | 568 | 77.0 | 0 | 0.0 |
| 2019 | 163 | 22.4 | 48 | 6.6 | 516 | 71.0 | 0 | 0.0 |
| 2020 | 100 | 16.7 | 107 | 17.9 | 391 | 65.4 | 0 | 0.0 |
| 2021 | 62 | 14.1 | 121 | 27.4 | 258 | 58.5 | 0 | 0.0 |
| 2022 | 52 | 17.9 | 83 | 28.5 | 150 | 51.5 | 6 | 2.1 |
| 2023 | 5 | 12.2 | 6 | 14.6 | 30 | 73.2 | 0 | 0.0 |
| Total | 1,275 | 29.6 | 378 | 8.8 | 2,646 | 61.5 | 6 | 0.1 |

IgE: immunoglobulin; IL: interleukin; TSLP: thymic stromal lymphopoietin

Percentage of patients within each year are shown.

**S-Table 5: IgE values (IU/mL) by OCS groups for all patients (as included in the paper) and for patients with negative SPT and SAT tests**

|  | **No OCS** | **iOCS** | **LTOCS** | **Total** | **iOCS vs LTOCS** |
| --- | --- | --- | --- | --- | --- |
|  | **N=215** | **N=2,330** | **N=1,760** | **N=4,305** | **P-value** |
| **All patients** | | | | | |
| **IgE, IU** | N=145 | N=1272 | N=778 | N=2,195 |  |
| Mean (SD) | 530.8 (796.5) | 494.7 (1747.6) | 397.2 (818.9) | 462.5 (1432.1) |  |
| Median (IQR) | 295 (121-608) | 206 (83-486) | 154 (53-389) | 190 (74-463) | <0.001 |
| <75, n(%) | 23 (15.9%) | 291 (22.9%) | 237 (30.4%) | 551 (25.1%) | <0.001 |
| >=75, n(%) | 122 (84.1%) | 981 (77.1%) | 541 (69.5%) | 1,644 (74.9%) |  |
| **Negative SPT/SAT*** | | | | | |
| **IgE, IU** | N=41 | N=219 | N=163 | N=423 |  |
| Mean (SD) | 305.3 (327.7) | 527.8 (3,457.6) | 288.2 (679.2) | 413.9 (2525.3) |  |
| Median (IQR) | 199 (90-359) | 140 (50-327) | 100 (40-248) | 132 (47-297) | 0.043 |
| <75, n(%) | 9 (22.0%) | 73 (33.3%) | 66 (40.5%) | 148 (35.0%) | 0.150 |
| >=75, n(%) | 32 (78.0%) | 146 (66.7%) | 97 (59.5%) | 275 (65.0%) |  |

*Patients with negative results for SPT or SAT and not positive on either test if both were taken

IgE: immunoglobulin E; iOCS: intermittent oral corticosteroid; IQR: interquartile range; LTOCS: long-term oral corticosteroid; OCS: oral corticosteroid; SAT: serum allergen-specific IgE test; SD: standard deviation; SPT: skin prick test

**S-Table 6: Combinations of high and low BEC and FeNO within each OCS group**

|  | No OCS | | Intermittent OCS^a^ | | Long-term OCS^b^ | |
| --- | --- | --- | --- | --- | --- | --- |
|  | N = 92 | | N = 799 | | N = 756 | |
| Low BEC / low FeNO, n (%) | 16 | (17.4) | 144 | (18.0) | 137 | (18.1) |
| Low BEC / high FeNO, n (%) | 12 | (13.0) | 133 | (16.7) | 204 | (27.0) |
| High BEC / low FeNO, n (%) | 21 | (22.8) | 149 | (18.7) | 97 | (12.8) |
| High BEC / high FeNO, n (%) | 43 | (46.7) | 373 | (46.7) | 318 | (42.1) |

BEC: blood eosinophil count; FeNO: fractional exhaled nitric oxide; OCS: oral corticosteroid

^a^ ≤90 days in last 12 months; ^b^ >90 days in last 12 months

Low BEC: <300 cells/µL

Low FeNO: <25 ppb

% within each OCS group are shown

**S-Table 7: phenotypic characterization of LTOCS users by LTOCS dose**

|  | **LTOCS daily dose,^a^ mg** | | | |  |
| --- | --- | --- | --- | --- | --- |
|  | **≤5**  **N=463** | **>5 - ≤10**  **N=610** | **>10 - ≤20**  **N=395** | **>20**  **N=219** | **P-value^g^** |
| **Gender**  Female, n (%) | 274 (59.2) | 370 (60.7) | 241 (61.0) | 138 (63.0) | 0.360 |
| **Age at biologic initiation, yrs**  Mean (SD)  18-34, n (%)  35-54, n (%)  55-79, n (%)  ≥80, n (%) | 54.7 (13.6)  42 (9.1)  168 (36.3)  243 (52.5)  10 (2.2) | 52.6 (14.3)  80 (13.1)  231 (37.9)  291 (47.7)  8 (1.3) | 52.7 (14.5)  52 (13.2)  149 (37.7)  190 (48.1)  4 (1.0) | 51.3 (14.0)  32 (14.6)  92 (42.0)  94 (42.9)  1 (0.5) | 0.004  0.002 |
| **BMI, kg/m^2^**  Median (IQR)  Underweight (<18.5), n (%)  Normal (≥18.5 - <25), n (%)  Overweight (≥25 - <30), n (%)  Obese (>30), n (%) | N=408  28 (24-32)  7 (1.7)  131 (32.1)  127 (31.1)  143 (35.0) | N=548  29 (25-33)  10 (1.8)  129 (23.5)  177 (32.3)  232 (42.3) | N=345  28 (24-33)  8 (2.3)  100 (29.0)  105 (30.4)  132 (38.3) | N=190  27 (23-33)  9 (4.7)  57 (30.0)  57 (30.0)  67 (35.3) | 0.956  0.969 |
| **Smoking status**  Current smoker, n (%)  Ex-smoker, n (%)  Never smoked, n (%) | N=381  12 (3.1)  131 (34.4)  238 (62.5) | N=525  14 (2.7)  161 (30.7)  350 (66.7) | N=331  9 (2.7)  101 (30.5)  221 (66.8) | N=184  5 (2.7)  57 (31.0)  122 (66.3) | 0.253 |
| **Asthma onset age, yrs**  Mean (SD)  < 18 yrs, n (%)  ≥18 yrs, n (%) | N=271  29.2 (19.4)  87 (32.1)  184 (67.9) | N=376  28.5 (19.2)  127 (33.8)  249 (66.2) | N=261  28.6 (19.4)  87 (33.3)  174 (66.7) | N=176  31.6 (17.9)  42 (23.9)  134 (76.1) | 0.288  0.168 |
| **Asthma duration, yrs**  Mean (SD) | N=264  26.5 (17.2) | N=364  24.3 (16.6) | N=258  23.5 (17.1) | N=174  21.1 (16.5) | 0.001 |
| Clinical characteristics | | | | |  |
| **Eosinophil phenotype^b^**  Grade 0: Non eosinophilic, n (%)  Grade 1: Least likely, n (%)  Grade 2: Likely, n (%)  Grade 3: Most likely, n (%) | N=423  0 (0.0)  0 (0.0)  25 (5.9)  398 (94.1) | N=555  0 (0.0)  0 (0.0)  41 (7.4)  514 (92.6) | N=354  0 (0.0)  0 (0.0)  42 (11.9)  312 (88.1) | N=197  0 (0.0)  0 (0.0)  11 (5.6)  186 (94.4) | 0.100 |
| **Allergen tests**  +ve SPT, n (%)  +ve SAT, n (%)  +ve SPT and/or +ve SAT, n (%) | N=148  79 (53.4)  N=80  48 (60.0)  N=174  94 (54.0) | N=93  55 (59.1)  N=73  36 (49.3)  N=138  73 (52.9) | N=89  50 (56.2)  N=42  27 (64.3)  N=119  69 (58.0) | N=103  49 (47.6)  N=41  25 (61.0)  N=123  60 (48.8) | 0.456  0.761  0.623 |
| **BEC, cells/µL**  Mean (SD)  Median (IQR) | N=326  552.9 (505.3)  400 (190-800) | N=449  417.4 (442.6)  300 (100-600) | N=307  442.9 (508.4)  300 (100-600) | N=154  667.2 (819.8)  435 (200-850) | 0.158 |
| **FeNO, ppb**  Mean (SD)  Median (IQR) | N=259  54.0 (48.5)  38 (22-69) | N=338  61.9 (58.6)  42 (20-85) | N=204  57.5 (52.6)  41 (18-80) | N=95  60.7 (56.4)  42 (24-76) | 0.724 |
| **Total IgE, IU**  Mean (SD)  Median (IQR) | N=235  399.0 (733.8)  154 (56-433) | N=235  342.6 (577.2)  145 (46-351) | N=173  440.2 (1,191.5)  151 (63-378) | N=99  475.8 (797.2)  200 (56-449) | 0.614 |
| **Exacerbations**  Mean (SD)  0, n (%)  1, n (%)  2, n (%)  3, n (%)  ≥4, n (%) | N=387  3.6 (4.8)  54 (14.0)  95 (24.5)  59 (15.2)  46 (11.9)  133 (34.4) | N=519  3.8 (4.4)  67 (12.9)  121 (23.3)  75 (14.5)  64 (12.3)  192 (37.0) | N=333  4.1 (4.3)  33 (9.9)  81 (24.3)  48 (14.4)  34 (10.2)  137 (41.1) | N=196  5.0 (6.6)  21 (10.7)  25 (12.8)  37 (18.9)  14 (7.1)  99 (50.5) | <0.001  <0.001 |
| **Asthma control^c^**  Well-controlled, n (%)  Partly controlled, n (%)  Un-controlled, n (%) | N=295  36 (12.2)  54 (18.3)  205 (69.5) | N=387  31 (8.0)  64 (16.5)  292 (75.5) | N=232  23 (9.9)  33 (14.2)  176 (75.9) | N=167  13 (7.8)  23 (13.8)  131 (78.4) | 0.028 |
| **Lung function^d^**  ppFEV_1_, mean (SD)  ppFEV_1_ <80%, n (%) | N=266  73.1 (24.2)  170 (63.9) | N=276  71.8 (24.5)  167 (60.5) | N=196  73.5 (23.5)  117 (59.7) | N=128  71.3 (23.8)  85 (66.4) | 0.729  0.940 |
| FEV_1_/FVC, median (IQR)  FEV_1_/FVC <0.7, n (%) | N=366  0.66 (0.56-0.75)  219 (59.8) | N=479  0.67 (0.57-0.75)  281 (58.7) | N=314  0.67 (0.56-0.75)  177 (56.4) | N=177  0.64 (0.55-0.73)  113 (63.8) | 0.421  0.901 |
| **Daily ICS dose,^e^ µg**  Mean (SD)  Median (IQR)  >0-125 µg, n (%)  >125-250 µg, n (%)  >250-500 µg, n (%)  >500-1000 µg, n (%)  >1000 µg, n (%) | N=143  435 (588)  250 (90-510)  43 (30.1)  29 (20.3)  33 (23.1)  30 (21.0)  8 (5.6) | N=214  548 (687)  368 (160-585)  45 (21.0)  37 (17.3)  58 (27.1)  54 (25.2)  20 (9.3) | N=164  527 (807)  335 (128-600)  41 (25.0)  30 (18.3)  40 (24.4)  39 (23.8)  14 (8.5) | N=59  596 (875)  184 (20-1000)  28 (47.5)  6 (10.2)  6 (10.2)  10 (16.9)  9 (15.3) | 0.972  0.912 |
| **Add on to ICS/LABA**  LAMA, n (%)  LTRA, n (%)  Theophylline, n (%)  Macrolide, n (%)  Cumulative OCS dose^f^ (mg), mean (SD)  Total OCS last yr^f^ (mg), mean (SD) | 129 (27.9)  129 (27.9)  31 (6.7)  22 (4.8)  594.1 (730.9)    2,029.8 (2,851.4) | 216 (35.4)  224 (36.7)  83 (13.6)  50 (8.2)  1,399.0 (1,710.9)  4,916.8 (6,571.3) | 133 (33.7)  143 (36.2)  55 (13.9)  36 (9.1)  2,239.7 (2,708.8)  8,099.7 (10,463) | 59 (26.9)  48 (21.9)  21 (9.6)  10 (4.6)  2,536.6 (1,855.5)  9,412.9 (7,299.5) | 0.588  0.961  0.024  0.263  <0.001  <0.001 |
| **Subsequent biologic**  Anti-IgE, n (%)  Anti-IL5/5R, n (%)  Anti-IL4Rα, n (%)  TSLP, n (%) | 104 (22.5)  328 (70.8)  30 (6.5)  1 (0.2) | 143 (23.4)  427 (70.0)  38 (6.2)  2 (0.3) | 107 (27.1)  253 (64.1)  35 (8.9)  0 (0.0) | 46 (21.0)  164 (74.9)  9 (4.1)  0 (0.0) | 0.539  0.946  0.605  0.345 |
| Healthcare resource utilization | | | | |  |
| **Asthma related ED visits**  Mean (SD)  0, n (%)  1, n (%)  2+, n (%) | N=387  0.5 (1.3)  307 (79.3)  31 (8.0)  49 (12.7) | N=535  0.5 (1.9)  443 (82.8)  38 (7.1)  54 (10.1) | N=324  0.6 (1.7)  256 (79.0)  28 (8.6)  40 (12.3) | N=172  1.6 (5.4)  116 (67.4)  18 (10.5)  38 (22.1) | 0.010  0.016 |
| **Asthma-related hospitalizations**  Mean (SD)  0, n (%)  1, n (%)  2+, n (%) | N=384  0.4 (1.4)  319 (83.1%)  36 (9.4%)  29 (7.6%) | N=536  0.5 (1.3)  433 (80.8%)  49 (9.1%)  54 (10.1%) | N=327  0.6 (1.5)  242 (74.0%)  40 (12.2%)  45 (13.8%) | N=174  1.2 (2.9)  121 (69.5%)  20 (11.5%)  33 (19.0%) | <0.001  <0.001 |

BMI: body mass index; ED: emergency department; ICS: inhaled corticosteroids; Ig: immunoglobulin; IL: interleukin; iOCS: intermittent oral corticosteroids; IQR: interquartile range; FEV_1_: forced expiratory volume in one second; FVC: forced vital capacity; LABA: long-acting β2-agonist; LAMA: long-acting muscarinic antagonist; LTRA: leukotriene receptor antagonist; LTOCS: long-term corticosteroids; ppFEV_1_: percent predicted forced expiratory volume in one second; SD: standard deviation; TSLP: thymic stromal lymphopoietin

^a^LTOCS >90 days in last 12 months

^b^ According to the expert consensus framework of Heaney et al, 2021^5^

^c^ Defined by GINA 2020 control test,^2^ asthma control questionnaire (ACQ)^3^ or asthma control test (ACT)^4^ (country specific). Conversion of ACT and ACQ to GINA control criteria as follows:

ACQ - Mean ACQ ≤0.75 = Well controlled; 0.75< Mean ACQ <1.5 = Partly controlled; Mean ACQ ≥1.5 = Uncontrolled

ACT - Total ACT >19 = Well controlled; 15< Total ACT ≤19 = Partly controlled; Total ACT ≤15 = Uncontrolled

^d^ For FEV_1_, post-bronchodilator measures were used if available, and pre-bronchodilator measures otherwise, while ensuring that pre- and post-biologic measures were both either pre- or post-bronchodilator. In the sub-population of patients included in the lung function analysis (N=1082), post-bronchodilator measurements were used for 70.9% patients.

^e^ Beclomethasone equivalent

^f^ Prednisone equivalent, last 90 days

^g^ Jonckheere–Terpstra test for trend with increasing LTOCS daily dose

**Supplemental Figures**

**S-Figure 1: subject disposition**

**
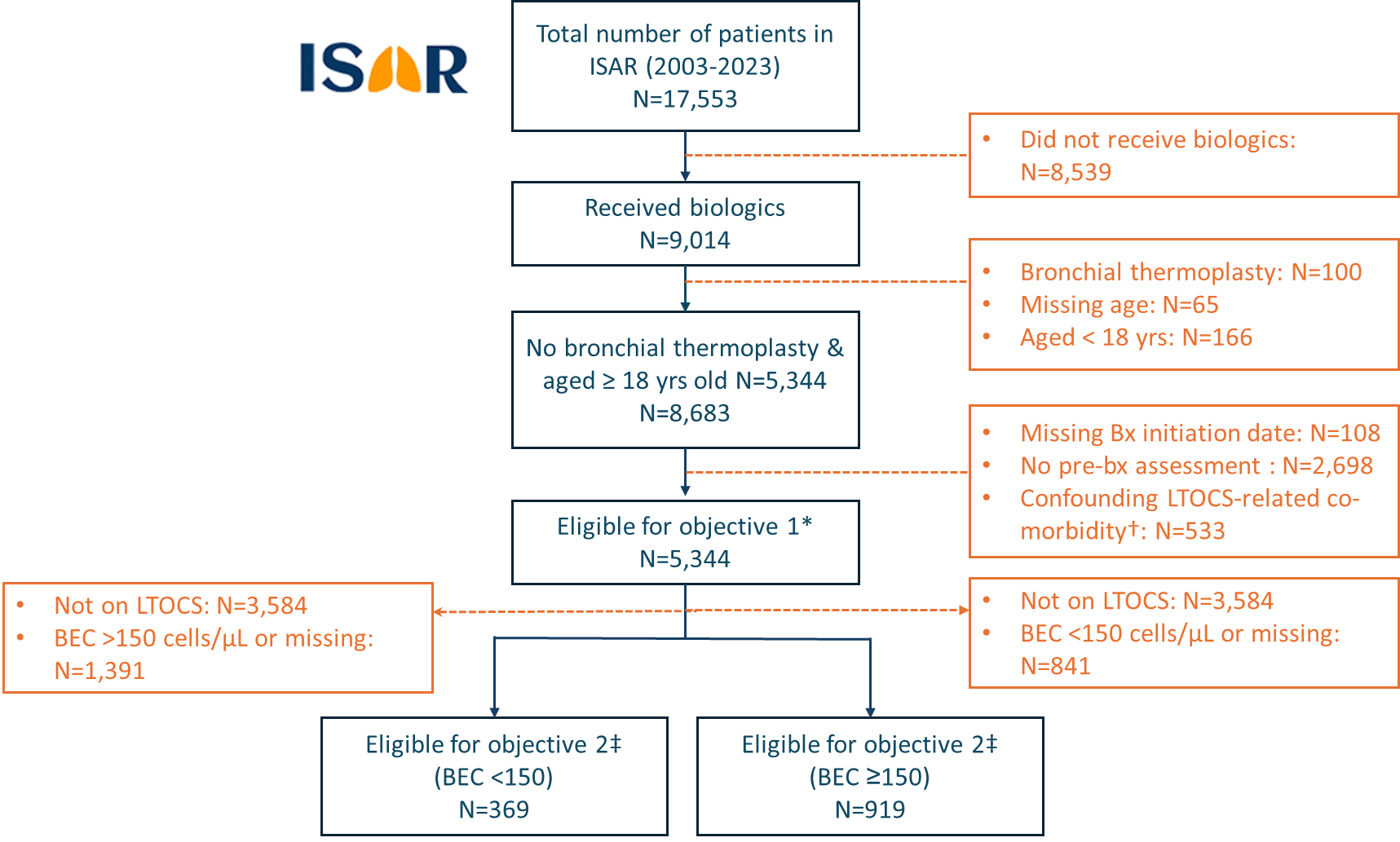
**

BEC: blood eosinophil count; ISAR: International Severe Asthma Registry; LTOCS: long-term oral corticosteroid
* To investigate the effect of OCS use prior to biologic initiation on severe asthma phenotype and biomarker profile
† Confounding LTOCS-related comorbidities
‡ To characterize the burden of disease among LTOC S users by biomarker profile

**S-Figure 2: BEC distribution of LTOCS users by LTOCS dose**


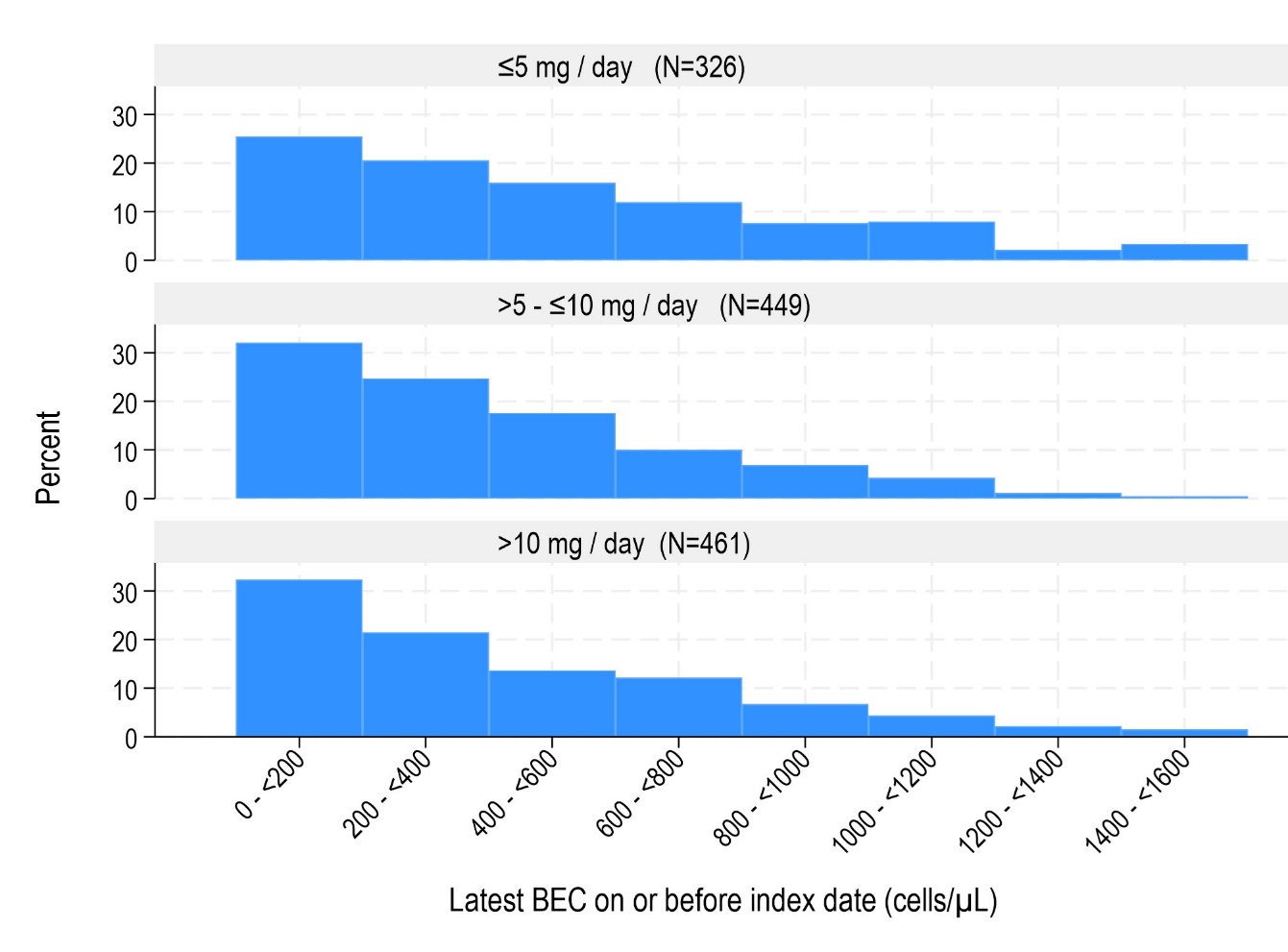
BEC: blood eosinophil count; LTOCS: long-term oral corticosteroid (i.e. use for >90 days)

Index date: date of biologic initiation

**References**

1. FitzGerald JM, Tran TN, Alacqua M, et al. International Severe Asthma Registry (ISAR): protocol for a global registry. *BMC Medical Research Methodology*. 2020;20(1):212.

2. Global Initiative for Asthma. Global Strategy for Asthma Managment and Prevention. Updated 2020. https://ginasthma.org/wp-content/uploads/2020/04/GINA-2020-full-report_-final-_wms.pdf [last accessed 18^th^ Nov 2024]

3. Juniper EF, O’Byrne PM, Guyatt GH, Ferrie PJ, King DR. Development and validation of a questionnaire to measure asthma control. *Eur Respir J*. 1999;14(4):902-907. doi:10.1034/j.1399-3003.1999.14d29.x

4. Nathan RA, Sorkness CA, Kosinski M, et al. Development of the asthma control test: a survey for assessing asthma control. *J Allergy Clin Immunol*. 2004;113(1):59-65. doi:10.1016/j.jaci.2003.09.008

5. Heaney LG, Perez de Llano L, Al-Ahmad M, et al. Eosinophilic and non-eosinophilic asthma: an expert consensus framework to characterize phenotypes in a global real-life severe asthma cohort. *Chest*. 2021;160(3):814-830.
